# Frameworks for supporting patient and public involvement in research: Systematic review and co‐design pilot

**DOI:** 10.1111/hex.12888

**Published:** 2019-04-22

**Authors:** Trisha Greenhalgh, Lisa Hinton, Teresa Finlay, Alastair Macfarlane, Nick Fahy, Ben Clyde, Alan Chant

**Affiliations:** ^1^ Nuffield Department of Primary Care Health Sciences University of Oxford Oxford UK; ^2^ North Central London Academic Foundation Programme London UK

**Keywords:** codesign, framework, hermeneutic review, patient and public involvement, systematic review

## Abstract

**Background:**

Numerous frameworks for supporting, evaluating and reporting patient and public involvement in research exist. The literature is diverse and theoretically heterogeneous.

**Objectives:**

To identify and synthesize published frameworks, consider whether and how these have been used, and apply design principles to improve usability.

**Search strategy:**

Keyword search of six databases; hand search of eight journals; ancestry and snowball search; requests to experts.

**Inclusion criteria:**

Published, systematic approaches (frameworks) designed to support, evaluate or report on patient or public involvement in health‐related research.

**Data extraction and synthesis:**

Data were extracted on provenance; collaborators and sponsors; theoretical basis; lay input; intended user(s) and use(s); topics covered; examples of use; critiques; and updates. We used the Canadian Centre for Excellence on Partnerships with Patients and Public (CEPPP) evaluation tool and hermeneutic methodology to grade and synthesize the frameworks. In five co‐design workshops, we tested evidence‐based resources based on the review findings.

**Results:**

Our final data set consisted of 65 frameworks, most of which scored highly on the CEPPP tool. They had different provenances, intended purposes, strengths and limitations. We grouped them into five categories: power‐focused; priority‐setting; study‐focused; report‐focused; and partnership‐focused. Frameworks were used mainly by the groups who developed them. The empirical component of our study generated a structured format and evidence‐based facilitator notes for a “build your own framework” co‐design workshop.

**Conclusion:**

The plethora of frameworks combined with evidence of limited transferability suggests that a single, off‐the‐shelf framework may be less useful than a menu of evidence‐based resources which stakeholders can use to co‐design their own frameworks.

## 1 BACKGROUND

It is a truth universally acknowledged by policymakers, researchers and research funding bodies that patients and the public should be “involved” in research, though there are different perspectives on what such involvement should look like and why it should happen. Other authors have summarized a diverse literature on this topic (see in particular a recent *BMJ* editorial^1^ and these theoretically informed reviews^2^, ^3^, ^4^, ^5^, ^6^, ^7^, ^8^). In sum, three main arguments prevail.

The first argument, described by some as normative and others as emancipatory, holds that patients have a right to have an input to research on their condition and that reducing the known power imbalances between researchers and patients is a moral duty of researchers, especially with oppressed and seldom‐heard groups.^2^, ^3^, ^4^, ^9^

The second, which some have described as consequentialist or efficiency‐oriented,^3^ is that patient and public involvement, by bringing a real‐world and lived‐experience perspective, improves the efficiency and value of research via a number of mechanisms: increasing its relevance to patients; improving recruitment and retention rates of research participants; extending the range of people represented in research studies; and improving dissemination of findings beyond academic audiences^6^, ^7^, ^10^, ^11^—though the evidence base for all these claims has been questioned.^10^, ^12^

The third argument is political and practical: that forming alliances with patients and the public is a defining feature of contemporary Mode 2 science (in which knowledge is co‐constructed by scientists and citizens, often beyond the walls of the university^13^); it increases the accountability and transparency of research and may be an effective way of attracting resources.^5^, ^10^

Notwithstanding the different (and to some extent incommensurable) perspectives represented by the above literature, it is clear that improving patient and public involvement in research is a high priority for research policymakers,^14^, ^15^, ^16^ research funders,^17^, ^18^, ^19^, ^20^ researchers,^21^, ^22^, ^23^ some academic journals^1^ and patient and lay organizations.^24^, ^25^, ^26^ Many of these groups have developed or are in the process of developing, structured frameworks, tools, guidelines and checklists in an attempt to improve their own performance and (in some cases) critique or assess the performance of others.

As a multi‐stakeholder research collaboration based in one of the UK's leading medical and biotechnology research regions, we had a strong commitment to strengthening patient and public input to our research. When we began this study, the UK National Institute for Health Research had recently put out for consultation its draft benchmarks for patient involvement in research.^27^ The conditions of our funding required us to report annually on our patient and public involvement activity. We sought, therefore, to identify one or more tools or frameworks that would help us support, evaluate, improve and report on the patient and public involvement work of research teams across our collaboration.

An initial browsing search identified numerous potential frameworks in both academic and grey literature, many of which appeared to have been carefully researched and some formally validated and field tested. Different groups had produced different frameworks, drawing on different principles, applying different theories and prioritizing different potential use cases. It was clear that developing a new framework from scratch was almost certainly unnecessary, but that the existing literature could benefit from a taxonomy and improved accessibility.

Accordingly, we set out to achieve three objectives. First, to identify, critically examine, summarize and synthesize existing tools, frameworks, benchmarks, guidelines and critical appraisal checklists for patient and lay involvement in research. Second, to determine which of the frameworks were actually used and why (and explain why others were not used). Third, to work with patient and lay groups and designers to adapt, simplify and annotate existing frameworks and improve their aesthetic appeal and usability. As the study unfolded (and for reasons explained in the results section below), this last aim evolved to incorporate a major focus on optimizing the *process* of running workshops aimed at generating and adapting and operationalizing frameworks for involving patients and lay people in research.

## 2 METHOD

### 2.1 Study design

Narrative systematic review, drawing on the principles of hermeneutic review,^28^ along with lay consultation and co‐design.^29^ Hermeneutic review consists of two interlinked cycles (described in more detail below): (a) accessing and interpreting the literature and (b) developing an argument. Searching is systematic but flexible and iterative. As sources accumulate, it becomes necessary to interpret, clarify and understand the emerging ideas and perspectives and to reject less relevant sources through progressive focusing. We have argued elsewhere that narrative review, which adds successive primary studies to an increasingly rich picture of a complex field of study, is the method of choice for synthesizing and making sense of a large and diverse body of primary literature where different groups of authors have approached the topic in very different ways.^30^

### 2.2 Data sources

We searched six databases (PubMed, Embase, Cinahl, Social Science Citation Index, Science Citation Index and PsycINFO) to end 2018 using the following concepts and key words (adapted from a strategy used by previous authors^31^): (a) consumer or community or patient or citizen or user or lay or public or stakeholder; (b) participate or engage or involve or consult or empower or collaborate or inform; (c) health or medical or biomedical or nursing; (d) research or evaluation; (e) tool or toolkit or framework or guideline or checklist. We hand‐searched eight journals (*Health Expectations*, *BMC Research Involvement and Engagement*, *International Journal of Consumer Studies*, *International Journal of Technology Assessment in Health Care*, *Health Research Policy and Systems*, *BMC Health Services Research*, *International Journal of Healthcare Quality Assurance* and *BMJ Open*) from January 2008 to December 2018.

We also searched selected grey literature sources (eg, guidance produced by national and international patient organizations and advocacy groups, health services or think tanks), collated sources already known to the authors and put out requests to our professional networks (including social media followers). When we identified papers that met our inclusion criteria, we checked the references of those papers and also put the title into Google Scholar to subsequent citations of it (an “ancestry and snowball” approach^32^). Where papers cited a specific theoretical underpinning, we obtained the original paper describing that theory. If a framework had been described in both academic and grey literature, we included only the former.

### 2.3 Inclusion and exclusion criteria

We included any published, systematic approach designed to inform, evaluate or report on patient and public involvement in health‐related research. There were no language restrictions. The main exclusion criteria were as follows: not a framework, not about research or lacking provenance (ie, unable to trace its source). We excluded frameworks that were focused only on communication or engagement (defined as explaining research to the public) as opposed to *involvement* (involving patients and the public in some way in planning, undertaking and disseminating research). Largely for practical purposes, grey literature was limited to publications from national or international organizations (eg, James Lind Alliance, INVOLVE, Canadian Institute for Health Research).

### 2.4 Data extraction and appraisal of quality

We used an Excel spreadsheet to summarize key aspects of each study (both theoretical and empirical). For each framework, data were extracted on the rationale for its development; provenance (including funding/sponsorship); patient/public input; theoretical basis (if any); orientation (initially using the taxonomy set out in the background above: “emancipatory,” “efficiency‐focused” and “practical,” and evolving as new categories emerged); fields and topics covered; format and accessibility; intended user(s) and purpose(s); examples of use; and critiques. Three reviewers (TG, AM and LH) undertook data extraction; each study was looked at by two reviewers with disagreements resolved by discussion. We attempted to contact lead authors of all papers to ask whether and by whom the framework had been used since its publication.

Using data from these domains, we applied the Canadian Centre for Excellence on Partnerships with Patients and the Public (CEPPP) evaluation tool, which assesses four aspects of a tool or framework^23^:

- scientific rigour (graded as 3 = good, 2 = moderate or 1 = weak);
- incorporation of patient/public perspective (graded as 3 = extensive, 2 = limited, 1 = absent or not reported);
- comprehensiveness (graded as 3 = good, covering all intended dimensions; 2 = limited, covering only some key dimensions; 1 = very limited); and
- usability (graded as 3 = good, extensive evidence of use beyond the study in which it was developed; 2 = emerging [for recently published frameworks with some evidence of use]; 1 = limited or unknown).

### 2.5 Analysis and synthesis of primary literature

Using the iterative hermeneutic methodology developed by Boell and Cecez‐Kecmanovic,^28^ we built an overall picture of the different kinds of frameworks and their strengths and limitations, adding detail and nuance as successive studies were incorporated.

As an example of our approach, our hand search turned up a paper by Staniszewska et al^33^ on the GRIPP1 (Guidance for Reporting Involvement of Patients and Public) framework for structuring how researchers report lay involvement in a clinical trial.^33^ Through citation tracking of that paper, we identified a number of additional “report‐focused” frameworks,^34^, ^35^, ^36^ including GRIPP2.^34^ Whilst we initially grouped all these as a subset of frameworks designed for planning and organizing patient involvement in research studies (“study‐focused”), detailed analysis revealed that these were separate categories with limited cross‐referencing between them.

We synthesized a preliminary set of resources based on the frameworks in our data set. To inform the practical workshops, rather than reproduce all the frameworks (since many covered similar ground), we worked with lay colleagues to select the “best‐in‐class” from different categories in our data set. In this process, we were guided by three questions: (a) did the framework score well using the CEPPP tool (see above)? (b) does it make sense to patients and lay people as well as researchers—and is it potentially usable by both? and (c) will it allow valid measurement and iterative improvement of patient and public involvement work by research teams?

### 2.6 Co‐design phase

We shared our preliminary set of 12 “best‐in‐class” resources in two preliminary 2‐hour development workshops attended by a total of 16 participants recruited from three local pre‐existing academic‐lay research partnerships (including researchers, patient involvement leads, patients, carers and advocates). We adapted the interactive and participatory methodology described by previous authors.^29^, ^37^ Prior to the first workshop, we made large‐scale diagrams of the different “best‐in‐class” frameworks from our systematic review and invited the groups to talk about them and use sticky notes to annotate them. We systematically captured and incorporated their suggestions for adaptation, and sought input from a professional design service to produce resources in multiple formats.

The workshop materials, suggested format, resources and facilitator notes produced in the two development workshops were refined through three further pilot workshops in contrasting clinical and research settings: a long‐established patient participation group for a specialist research group in blood disorders; a recently established lay partner group for a community‐based mental health research programme; and an academic‐lay‐industry partnership seeking to establish working principles and evaluation methods for lay participation in industry‐led clinical trials. Full details of these workshops will be presented in a separate paper.

## 3 RESULTS

### 3.1 Description of data set

**Figure 1:**
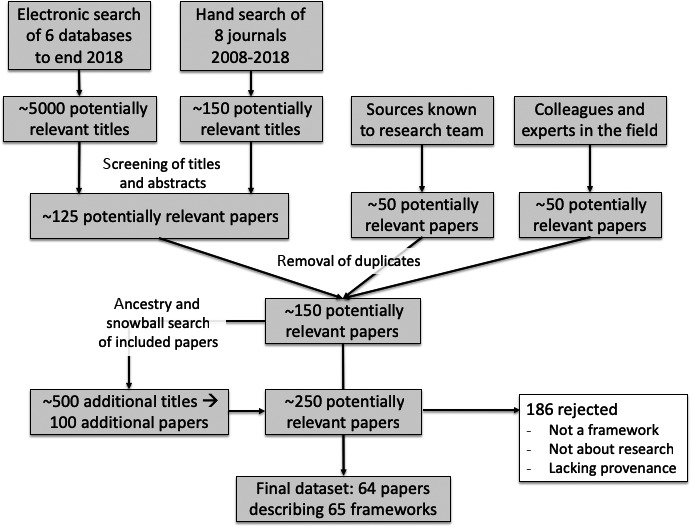
Study flow chart

The study flow chart is shown in Figure 1. Of over 5000 titles, 150 papers were retrieved in full text; this sample was extended to 250 using ancestry and snowball searches. After applying exclusion criteria, our final data set consisted of 64 papers describing 65 frameworks from 10 countries (one paper described two frameworks^35^): UK (34 papers^5^, ^12^, ^27^, ^33^, ^34^, ^35^, ^36^, ^38^, ^39^, ^40^, ^41^, ^42^, ^43^, ^44^, ^45^, ^46^, ^47^, ^48^, ^49^, ^50^, ^51^, ^52^, ^53^, ^54^, ^55^, ^56^, ^57^, ^58^, ^59^, ^60^, ^61^, ^62^, ^63^, ^64^), United States (14 papers^10^, ^65^, ^66^, ^67^, ^68^, ^69^, ^70^, ^71^, ^72^, ^73^, ^74^, ^75^, ^76^, ^77^), Canada (7 papers^20^, ^78^, ^79^, ^80^, ^81^, ^82^, ^83^), Netherlands (3 papers^84^, ^85^, ^86^) and one paper each from Australia,^31^ Spain,^87^ Zambia,^88^ a WHO consortium led from Switzerland,^89^ a Southern African consortium led from South Africa^90^ and a European consortium led from Belgium.^91^

The included publications described toolkits, tools, frameworks, checklists, benchmarks or maps for informing, guiding, assessing or reporting on patient and/or public involvement in research. A total of 56 frameworks were written up in 55 academic papers.^5^, ^10^, ^12^, ^31^, ^33^, ^34^, ^35^, ^36^, ^38^, ^39^, ^40^, ^41^, ^42^, ^43^, ^44^, ^45^, ^47^, ^48^, ^49^, ^50^, ^52^, ^53^, ^55^, ^56^, ^57^, ^60^, ^61^, ^62^, ^63^, ^64^, ^65^, ^66^, ^67^, ^69^, ^70^, ^71^, ^72^, ^73^, ^74^, ^75^, ^76^, ^77^, ^78^, ^79^, ^80^, ^81^, ^82^, ^83^, ^84^, ^87^, ^88^, ^89^, ^90^, ^92^ Of these, 44 were available open access. Nine frameworks were in the grey literature, all of which were publicly available.^20^, ^27^, ^46^, ^51^, ^54^, ^58^, ^68^, ^85^, ^91^

The data extraction and scoring spreadsheet for the 65 frameworks is available from the authors. Almost all frameworks in our sample scored moderately or very highly on the CEPPP tool for scientific rigour (our scoring acknowledged a wide range of study designs). Most frameworks had been developed using a systematic approach with substantial input from patients or lay people, though approaches used varied considerably. Some groups had used primary qualitative research^50^, ^55^, ^56^, ^57^, ^81^, ^84^ and/or qualitative, thematic or narrative literature review,^5^, ^10^, ^33^, ^34^, ^35^, ^39^, ^40^, ^41^, ^42^, ^44^, ^47^, ^58^, ^62^, ^63^, ^64^, ^70^, ^74^, ^76^, ^81^, ^82^, ^85^, ^87^ realist review (asking “what works for whom in what circumstances”),^48^, ^79^, ^80^ a consensus‐building process such as Delphi^34^, ^38^, ^52^, ^58^, ^69^ or economic modelling.^71^ Other frameworks had been developed in a more pragmatic way by working groups (typically involving lay people, researchers and/or research funders) with extensive consultation but without an in‐depth review of the relevant academic literature.^20^, ^27^, ^36^, ^59^, ^69^, ^78^, ^91^ Some groups used a combination of literature review, qualitative research and workshops.^31^, ^34^, ^53^, ^58^, ^65^, ^72^, ^73^, ^75^, ^77^, ^80^, ^81^, ^82^, ^85^, ^88^, ^89^ Some covered all lay involvement; others were restricted to specific groups such as older people,^61^, ^80^ those with a specific clinical condition,^36^, ^59^, ^72^, ^75^, ^84^, ^87^ those with or at risk of a genetic condition^72^, ^85^ or underserved or marginalized groups.^65^, ^69^, ^76^, ^77^, ^79^ Most grey literature frameworks gave limited details of methodology, though one drew on academic sources^91^ and two described and referenced a literature review.^46^, ^54^

Four papers proposed a “framework of frameworks” taxonomy of approaches to patient and public involvement in research (see Discussion for details).^62^, ^63^, ^64^, ^83^ The remaining 61 frameworks could be grouped into five main categories (though several had features of more than one):

1. Power‐focused: designed to surface, explore and overcome researcher‐lay power imbalances;
2. Priority‐setting: designed to involve patients and lay people in setting research priorities;
3. Study‐focused: designed to maximize recruitment and retention to clinical trials (and, less commonly, other study designs), thereby improving the quality and efficiency of research and/or maximizing its societal impact;
4. Report‐focused: designed to guide writing up and critical appraisal of research reports;
5. Partnership‐focused: designed to assure transparency and public accountability in researcher‐lay collaborations.

In the first and last of these, the presumed unit of analysis was a partnership (actual or desired). In the middle three, the presumed unit of analysis was a research study (usually, a clinical trial). In Table 1, we summarize the features of the five categories of framework, highlighting the ones we selected as “best in class” (high score on CEPPP tool and liked by our patient advisers). Below, we describe the frameworks in each category in more detail, giving one example of each. The other “best in class” frameworks are reproduced in the Appendix S1.

**Table 1:** Taxonomy of frameworks for supporting and evaluating patient and public involvement in research

Category with selected “best in class” examples	Main focus of frameworks in this category	Comment
**Power‐focused frameworks** Oliver et al^44^ Morrow et al^42^ Gibson et al^40^ Gradinger et al^41^ Belone et al^65^	Conceptualizing, surfacing and challenging power differentials between researchers and patients/lay people. Ethical principles for such power‐sharing. Community‐based participatory research (CBPR) applies a power‐focused lens to researching marginalized or seldom‐heard communities	Tend to be academically led, richly theorized and emancipatory in ethos. They have informed and underpinned more pragmatic, partnership‐focused frameworks developed subsequently
**Priority‐setting frameworks** Viergever et al^89^ Pollock et al^36^	Principles and methods for involving patients and lay people in setting research priorities. Includes using a structured and transparent process; ensuring diversity of participants; providing background evidence; involving technical and topic experts; and translating priority issues into researchable questions	James Lind Alliance (UK) and Patient‐Centered Outcomes Research Institute (USA), for example, promote priority‐setting partnerships between researchers and lay people
**Study‐focused frameworks** Evans et al^48^ Shippee et al^70^ NIHR Research Design Service^51^ Dillon et al^73^	Principles and methods for involving patients and lay people in conducting research, especially trials. They follow the research cycle from grant application to disseminating findings and achieving impact. Most cover building a culture of involvement, attending to local context, input from a senior leader, developing relationships and trust, ensuring representativeness, training and capacity‐building, and facilitation	Most study‐focused frameworks include limited theory but Evans et al, for example, use a realist approach to explore link between context, mechanism and outcome
**Report‐focused frameworks** Stanislavska et al^34^ Pollock et al^53^	Reporting guidelines for writing up how patient and public involvement was approached in a research study	Stanislavska addresses primary research (eg, trials); Pollock addresses systematic reviews
**Partnership‐focused frameworks** Boote et al^38^ Baines et al^60^ INVOLVE^54^ De Wit et al^84^ Canadian Institute of Health Research^20^ Patient‐Focused Medicines Development^91^	Particular emphasis on demonstrating what measures are in place to support the academic‐lay partnership and provide an audit trail to account for its activities. Focus is on governance structures (eg, co‐chairing), public release of data (transparency), communication processes (eg, showing that researchers have responded to comments) and training (of both researchers and patients)	Frameworks in this category tend to link a specific value or principle with a particular set of metrics of involvement and impact

**Figure 2:**
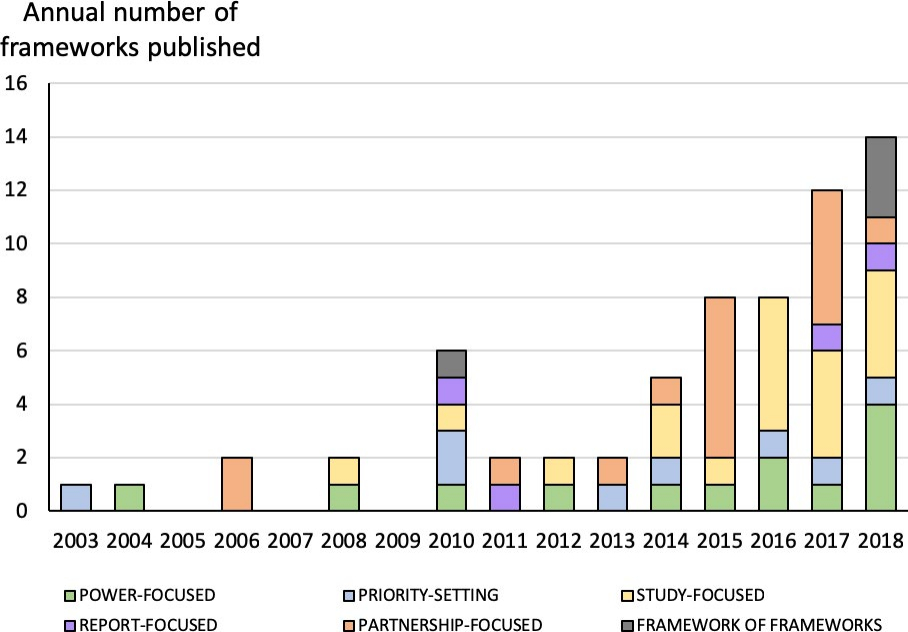
Number of frameworks on patient and lay involvement in research published annually (includes academic and grey literature)

The number of publications per year in our sample is shown in Figure 2. Despite there being no date limit on our database search, no frameworks had been published before 2003. Since then, the number published annually has grown steadily. There has been a recent steep rise in the publication of study‐focused and partnership‐focused frameworks and (in 2018) reviews of frameworks.

### 3.2 Power‐focused frameworks

Thirteen frameworks (eight from UK^5^, ^39^, ^40^, ^41^, ^42^, ^43^, ^44^ and five from United States^65^, ^66^, ^67^, ^76^, ^77^) were developed by academically led teams whose primary interest was studying and challenging power differentials in researcher‐lay partnerships. They applied theories from critical sociology such as Foucault (who proposed that knowledge and power are intimately related), Habermas (who explored the concealed power games between those based in the “system” and those outside it) or Bourdieu (who wrote about different kinds of social and cultural capital, of which specialized knowledge is one component), or from critical public health (notably, theories of power‐sharing in community‐based participatory research).

Whilst power‐focused frameworks addressed similar domains to those in other categories in our taxonomy, they asked more radical questions. They were particularly interested, for example, in surfacing power imbalances, values and hidden motives. For example, they did not merely talk about “empowering” patients and lay people; they asked questions such as “who gets to define what empowerment is?” and “whose interests are served by so‐called empowerment?”

The earliest power‐focused framework in our data set was led by Oliver's group at the Institute of Education. It was published in 2004^44^ and updated in 2008^39^ (see Appendix S1 for diagram). Drawing on Arnstein's ladder of participation, they rated lay input on a continuum from none to consultation to collaboration to control^93^; they also used Mullen's distinction between proactive and reactive behaviour by researchers (researchers could invite lay groups, invite individuals, respond to lay action or do little or nothing).^94^ Oliver et al^5^ subsequently updated and extended this framework further to include drivers for involving patients and public (why researchers invite involvement; why people get involved); processes of involvement (how people are brought together, how they interact); and the impact of involvement (public engagement with and/or influence on science).

Morrow et al^42^ developed a Quality Involvement Framework based on Foucauldian notions of power and depicting both a user perspective—what was the individual *able to do* (eg, access resources); what could they *potentially do* (eg, apply for a role); and what did they *feel* (eg, valued, empowered, conscious of power dynamics)—and a corresponding research context perspective—comprising research relationships, ways of doing research and research structures (see Appendix S1 for full questionnaire).

Prainsack, whose theoretical starting‐point was the “opening‐up” of science proposed by sociologists of science such as Nowotny,^95^ worked with various genetics alliances to produce a set of six principles for genetic research; many of the questions are framed explicitly in terms of power (“who sets the agenda?”; “by whom is it decided what good outcomes are?”; “who has access to what data?”).^43^

Power‐focused frameworks exploring the values and ethical principles of lay involvement in research (see examples in Appendix S1)^40^, ^41^, ^45^ appear to have informed the subsequent development of more pragmatic, partnership‐focused frameworks (discussed below).

Some publications addressed researcher‐community power differentials through the lens of community‐based participatory research,^65^, ^66^, ^67^, ^76^ including a comprehensive framework synthesized from earlier literature by Belone et al^65^ (reproduced in Appendix S1). This considered contexts (eg, socio‐economic, policy, institutional, historical), group dynamics (structural, individual and relational), the nature of the intervention and/or research (eg, cultural fit, partnership synergy, appropriateness of study design) and outcomes (in relation to both individual and community health and the wider system, including capabilities, power relations and “cultural renewal”).

Two recent frameworks were published from the US Patient‐Centered Outcomes Research Institute (PCORI), an arms‐length government organization (and leading funder of patient involvement research) whose main goal is ensuring that comparative effectiveness studies address outcomes relevant to patients. One paper described a framework for extending such research with the principles of community‐based participatory research, with a view to building relationships with underserved communities.^76^ This framework emphasizes using assets‐based rather than deficit models to assess and extend community capacities and embracing anthropological as well as biomedical perspectives on the causes and management of illness. The other paper described a power‐focused framework for guiding the involvement of poor and underserved populations in research using routinely collected data from patient health records.^77^

### 3.3 Priority‐setting frameworks

Eight frameworks, from Canada,^78^ Netherlands,^86^ Switzerland,^89^ UK,^36^, ^46^ Zambia^88^ and United States,^68^, ^69^ summarized guidance for a structured process to help ensure that patients and lay people are involved (along with clinicians and researchers) in deciding which topics to prioritize for future research.

In 2003, Lomas et al^78^ published the output of a Canadian‐UK health services research collaboration. Based on two extensive consultation exercises in the respective countries, they proposed a six‐step approach: identify stakeholders; identify and assemble any data needed; design and complete the consultation, bringing together lay partners as well as people with knowledge (technical working group) and people with power (decision‐makers); validate the identified priority issues against other sources of similar information; translate priority issues into researchable topics and themes; and return to validate the priority research themes with stakeholders.

These six steps were refined and extended in a later synthesis by Viergever et al,^89^ oriented primarily to public health and health systems research in low‐ and middle‐income countries. They added two preliminary steps (understand the national and local context, and decide whether a comprehensive approach is needed at all), efforts to include the voices of marginalized groups, and included a follow‐up evaluation (see full framework in Appendix S1).^89^ More recently, researchers from Zambia published a similar health system‐oriented framework based on a systematic analysis of previous frameworks and two user workshops.^88^

**Figure 3:**
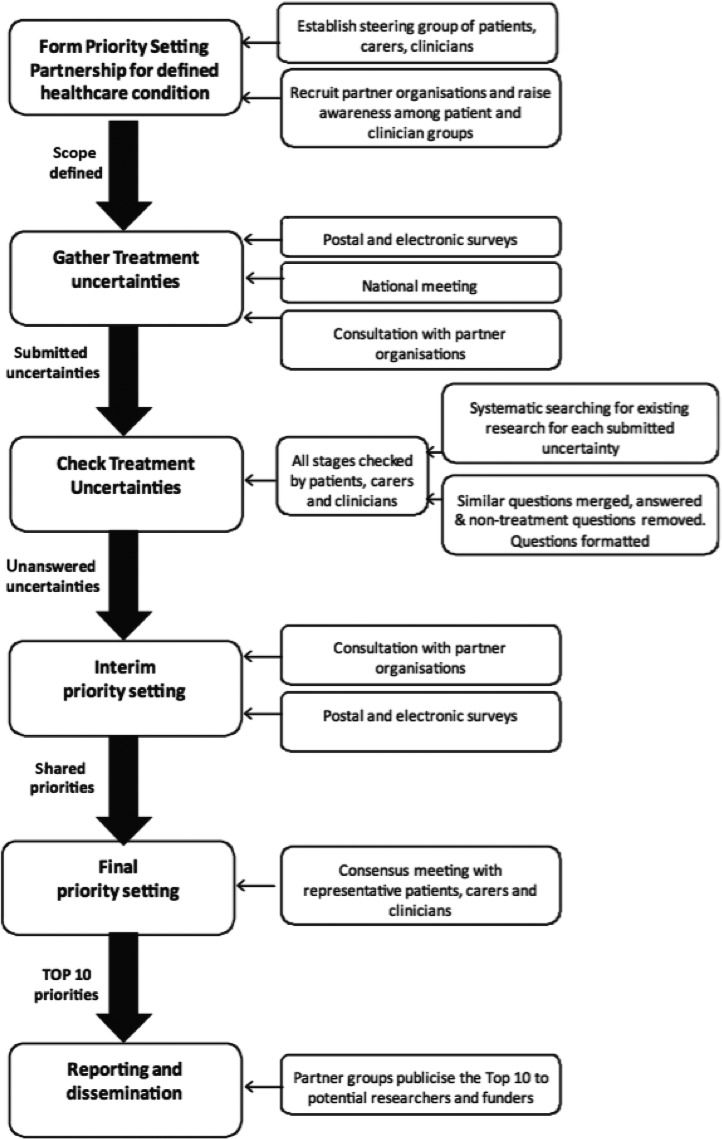
Example of framework for patient and lay involvement in research priority‐setting, reproduced with permission from Pollock et al^36^

The UK‐based James Lind Alliance developed a framework for topic‐focused priority‐setting partnerships oriented mainly to the design of new clinical trials in 2008 and updated in 2013 (Figure 3).^36^ They emphasized five principles: transparency of process; balanced inclusion of patient, carer and clinician interests and perspectives; exclusion of non‐clinician researchers for voting purposes (they may be involved in all other aspects of the process); exclusion of those with significant competing interests, for example pharmaceutical companies; and maintained audit trail. A similar framework, oriented to priority‐setting in comparative effectiveness research, was produced by PCORI in the United States.^68^

Pollock et al^36^ adapted the James Lind Alliance methodology to increase participation by a potentially excluded group (stroke patients with aphasia). Modifications included visits to individuals' homes; visits to patient advocacy and support groups; provision of materials in multiple formats including easy‐read and aphasia‐friendly; assistance with responding (eg, scribing); and assistance with access to venues.

Unique among our data set was a Dutch framework describing what the authors called the Dialogue Model, which used participatory (and explicitly power‐sharing) methods to set research priorities, including an early consultation phase to “enable patients to develop their own voice and agenda [and prepare] for broader collaboration with other stakeholder groups” (page 160).^86^

Whilst the James Lind Alliance drew on the principles of power‐sharing developed by Oliver et al,^44^ critical social scientists have suggested that despite the democratic intentions of its architects, priority‐setting partnerships do not necessarily empower patients, since researchers retain—and may choose to wield—the power to define what a legitimate research question is and how to answer it.^96^, ^97^

### 3.4 Study‐focused frameworks

Of 19 frameworks in this category (from UK,^12^, ^35^, ^47^, ^48^, ^49^, ^50^, ^51^, ^52^ United States,^70^, ^71^, ^72^, ^73^, ^74^, ^75^ Canada,^79^, ^80^, ^81^ Spain^87^ and Southern Africa^90^), 14 were based on a more or less linear model of a clinical trial and proposed how patient and lay involvement could be woven into it at every stage from writing the proposal to disseminating the findings.^12^, ^35^, ^47^, ^48^, ^51^, ^70^, ^72^, ^73^, ^75^, ^79^, ^80^, ^81^, ^87^, ^90^ One framework focused on the phase before formal ethical approval was gained^50^ and one on the involvement of patients in setting clinical outcomes.^74^ One considered the economic costs and benefits of lay involvement in different phases of a clinical trial^71^; and one addressed how to maintain recruitment to successive trials over time.^49^ A framework for improving patient engagement in Alzheimer's disease trials highlighted specific challenges with this target population and offered solutions based on a literature review.^87^

**Figure 4:**
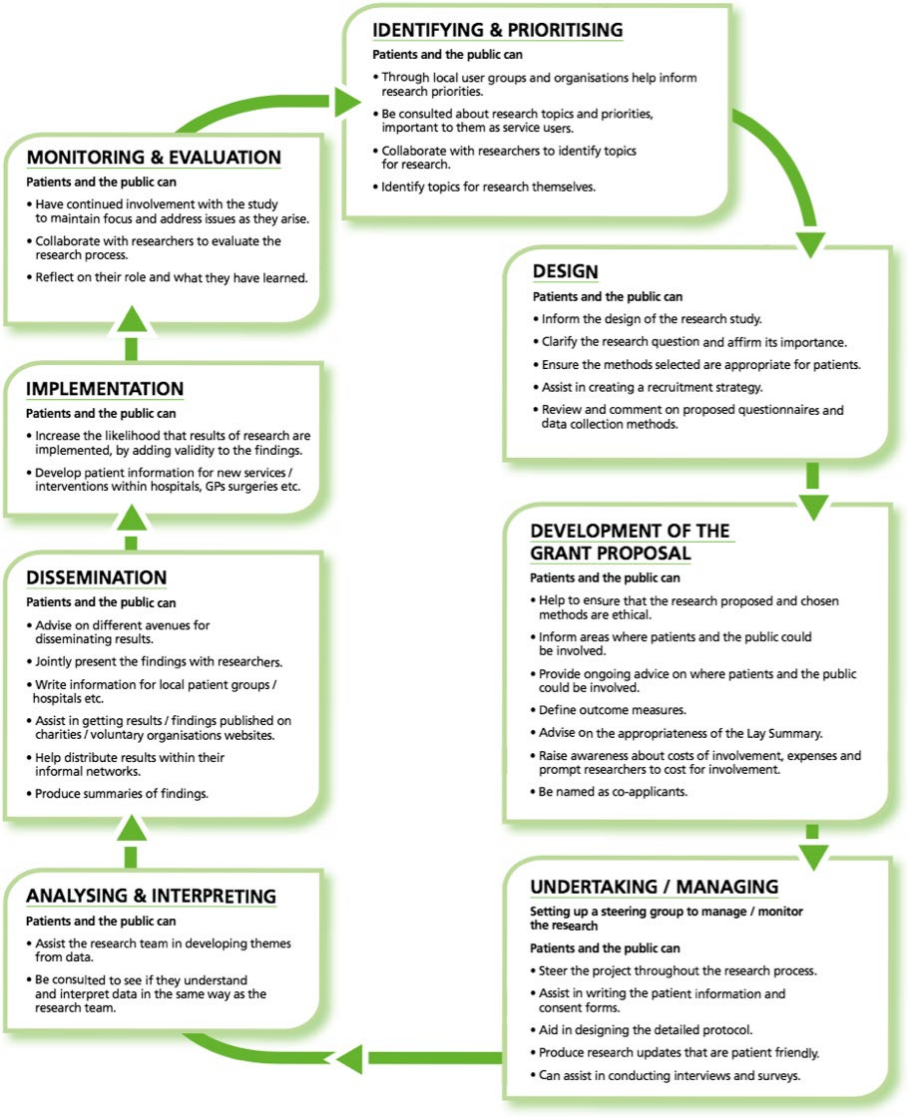
Example of study‐focused framework for patient and lay involvement in research, reproduced with permission from the NIHR Research Design Service^51^

Most studies in this category were funded by bodies that sponsor clinical trials and/or seek to ensure patient input to such trials. These include the UK National Institute for Health Research (NIHR) Research Design Service (Figure 4),^51^ PCORI in United States,^70^ and international development funders.^90^

All publications in this category emphasized that, in the view of the authors, patient and lay input throughout a clinical trial would make the trial more relevant, more appealing to potential participants, more likely to reach its target recruitment, more likely to retain participants and more likely to generate and disseminate high‐quality research knowledge.

Whilst study‐focused frameworks differed in detail, common features included the need to: (a) assess and understand the local context and nature of the proposed study; (b) plan ahead and resource each step adequately; (c) go beyond tokenism (eg, ensuring that patient involvement is more than “ticking a box”); (d) address inclusivity (eg, by developing research capacity in satellite clinics serving ethnically diverse sub‐populations); (e) address human aspects (building relationships, clarifying roles, communicating clearly, establishing trust and sharing information); and (f) develop and nurture an *ongoing* relationship with lay partners (anticipating and transitioning to the next trial). Some offered tools to work systematically through procedural and process aspects of patient and lay involvement (eg, what to write on official forms and where to submit them).

One paper proposed a set of “ethical” questions to ask about user involvement in relation to a clinical trial^35^: Are users fully informed about the proposed study?; Are they able to opt out?; Are they well enough to participate?; Are they overcommitted with other research?; How will their details be kept?; Will their expenses be met?; Will they become distressed by taking part?; and Will they receive peer supervision and/or peer support?

Two studies (from UK^48^ and Canada^80^) had used realist methods to explore the links between context, mechanism and outcome in patient involvement activities linked to clinical trials; an example is shown in the Appendix S1. Both found that effective, non‐tokenistic involvement of lay people in clinical trials depended on the interaction between contextual factors (nature of the research field, leadership by the principal investigator, a culture of involvement) and mechanisms (notably, a senior member of the team leading on lay involvement, nurturing of interpersonal relationships and development of mutual trust, facilitation and feedback).^48^, ^80^

Another paper proposed an economic model for estimating the financial value of patient involvement in the clinical development of oncology drugs.^71^ The authors used an economic technique (expected net present value) for assessing cost and benefits in drug development (based on five key drivers: revenue, costs, time, risk and intangibles). They applied this in a novel way to patient engagement in the research process. They found that more patient involvement substantially lowers the chances (and hence the cost) of protocol amendments and also improves the participant experience, leading to fewer withdrawals from the study (again, with major predicted cost savings).

**Figure 5:**
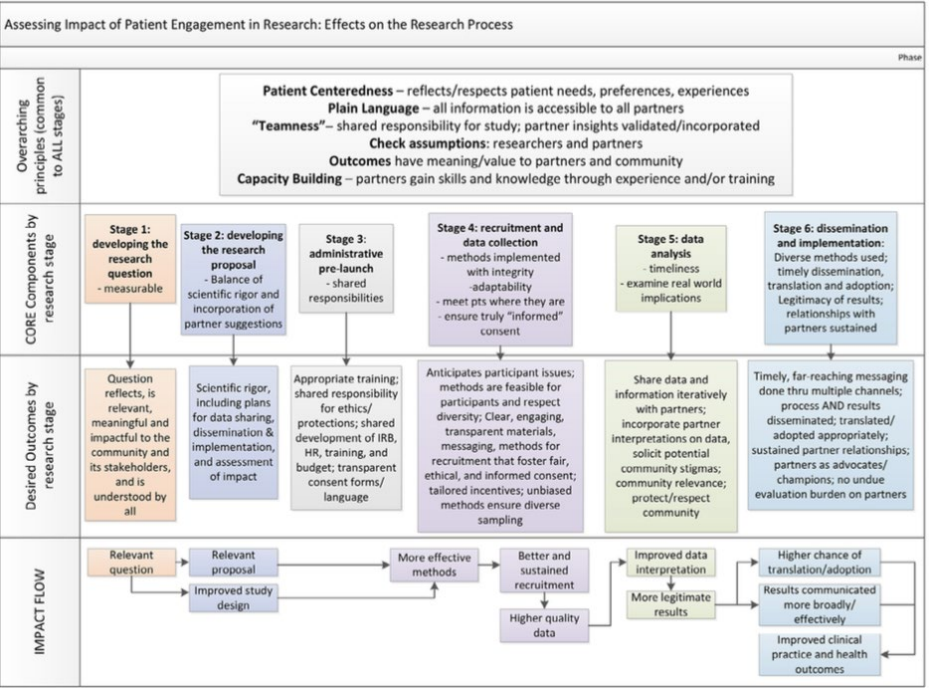
Example of study‐focused framework for measuring the impact of patient and lay involvement in research, reproduced under creative commons licence from Dillon et al^73^

Two recently published study‐focused frameworks included an additional dimension of measuring the impact of such involvement.^68^, ^72^ Dillon et al,^73^ for example, used a literature review along with user workshops to develop the Critical Outcomes of Research Engagement (CORE) framework shown in Figure 5, through which key aspects of patient and lay involvement can be tied to specific and measurable outcomes (see Appendix S1 for a table of specific metrics). For example, asking patients to feed back on the wording of questionnaire items (process) would be expected to increase the completion rate (outcome) and hence the robustness of the findings (impact).

### 3.5 Report‐focused frameworks

Four frameworks, all from UK, offered a checklist for critically appraising a published study for the quality and comprehensiveness of patient and lay involvement. Three covered primary studies^33^, ^34^, ^35^; and one covered systematic reviews.^53^ All addressed (at least in broad outline) the structure of a clinical trial report (eg, rationale, methodology, findings, discussion, evaluation or reflection) or systematic review equivalent. Two were produced by the GRIPP team as part of the EQUATOR network; the latest version is the 34‐item long‐form GRIPP‐2 checklist (shown in short form in Table 2).^34^ Report‐focused frameworks for primary studies assumed that the research design was a clinical trial; they addressed the same elements in broadly the same way as study‐focused frameworks, but did so retrospectively (as a quality checklist) rather than prospectively (to guide activity).

**Table 2:** Example of report‐focused framework: GRIPP2 short form

Section and topic	Item
1. Aim	Report on the aim of PPI (patient and public involvement) in the study
2. Methods	Provide a clear description of the methods used for PPI in the study
3. Study results	Outcomes—report the results of PPI in the study, including both positive and negative outcomes
4. Discussion and conclusions	Outcomes—comment on the extent to which PPI influenced the study overall. Describe positive and negative effects
5. Reflections/critical perspective	Comment critically on the study, reflecting on the things that went well and those that did not, so others can learn from this experience

Reproduced under Collective Commons Licence 4.0.

### 3.6 Partnership‐focused frameworks

Seventeen frameworks (from United States,^10^ Canada,^20^, ^82^ Australia,^31^ UK,^27^, ^54^, ^55^, ^56^, ^57^, ^58^, ^59^, ^60^, ^61^ the Netherlands^84^, ^85^ and Belgium^91^) were classified as predominantly partnership‐focused, in that they were explicitly designed to optimize collaborative partnerships between researchers and lay people or lay organizations and measure key dimensions of partnership success—preferably quantitatively and reproducibly. Most such frameworks placed particular emphasis on governance, public release of data (transparency) and accountability. Details of some exemplar partnership‐focused frameworks are listed in the Appendix S1.

The James Lind Alliance (described above in the “Priority‐setting” category above) was one of the first groups to propose some core principles underpinning research partnerships with patients and the public: transparency, balance, exclusion of conflicts of interest, and audit.^46^

Boote et al^38^ in 2006 used an extensive Delphi process to generate eight principles (including agreed roles, reimbursement, respect and training), each with an audit indicator, for supporting researcher‐lay partnerships more generally (see Appendix S1 for details). These early initiatives are typical of approaches that seek to deliver what Daniels has termed “accountability for reasonableness” in the public sector—that is, demonstrating a systematic, transparent and auditable process through which citizens and service users can contribute to, and help oversee, the work of a public body.^98^

A number of academic‐lay partnerships have produced similar frameworks, typically as a result of hybrid funding from academic, service and patient organizations.^31^, ^41^, ^55^, ^59^, ^60^, ^61^, ^77^, ^79^, ^82^, ^84^, ^85^, ^91^ Common themes in this category included governance mechanisms including formal power‐sharing arrangements (eg, co‐chairing); good leadership and project management; clear and effective communication (including commitment to listening and responding); mechanisms to ensure inclusivity (eg, outreach, reimbursement); training and capacity‐building (of both researchers and lay partners); regular activities to maintain contact; promotion of shared values and collaborative learning (what one framework called a “participatory culture”^82^); and metrics for measuring processes and impact.

The above themes featured prominently, for example, in a UK‐based consortium's “UK PPI Standards for public involvement in research” (inclusive opportunities, working together, support and learning, communications, impact and governance), published in March 2018.^27^ These six standards were produced by a partnership between NIHR, Health Research and Care Wales, Chief Scientist Officer Scotland and the Public Health Agency in Northern Ireland, and based on extensive engagement work with almost 700 participants including patients, the public and researchers. Each standard is accompanied by a set of auditable metrics, all addressed at individual, team and organizational level, on which NIHR‐funded research organizations are invited to report. The INVOLVE principles are reproduced in Table 3, and the standards are reproduced in full in the Appendix S1.

**Table 3:** Example of partnership‐focused framework: the involve values and principles framework

Values	Summary principles	Example of measurable impact
1. Respect	Researchers, research organizations and the public respect one another's roles and perspectives	Public members' contributions are acknowledged, for example as co‐applicants in research applications, as authors or co‐authors of publications, or as presenters or co‐presenters of research findings (1e)
2. Support	Researchers, research organizations and the public have access to practical and organizational support to involve and be involved	Public members' expenses are covered, and they are informed in advance if payment will be offered for their time (2d)
3. Transparency	Researchers, research organizations and the public are clear and open about the aims and scope of involvement in the research	Clear information is given about public members' role and what has been agreed; information is given about the time period and type of contribution (eg, partnership, advisory role, reviewer) (3b)
4. Responsiveness	Researchers and research organizations actively respond to the input of public members involved in research	Public members are listened to and changes are made to the research as a result of the insights, advice and guidance received; where changes are not made, reasons are explained (4b)
5. Fairness of opportunity	Researchers and research organizations ensure that public involvement in research is open to individuals and communities without discrimination	The diversity required for the research is considered and an effort is made to involve those who reflect that diversity (5a)
6. Accountability	Researchers, research organizations and the public are accountable for their involvement in research and to the people affected by the research	At the end of a research study, all those who have worked together actively reflect on the public involvement in the project and assess the learning and how it has gone; everyone is given an opportunity to feed back about their experience of involvement (6d)

Reproduced with permission of INVOLVE. Numbers in column 3 refer to paragraphs in INVOLVE document.

### 3.7 Evidence of framework use

The only dimension of the CEPPP tool on which a high proportion of frameworks scored poorly was usability (which we interpreted to include actual evidence of use). Power‐focused frameworks were rarely used directly, but they informed and underpinned subsequent work on more applied categories of framework.^46^, ^51^, ^58^ Some but not all priority‐setting,^46^, ^78^, ^85^ study‐focused^79^, ^80^, ^81^ and partnership‐focused^31^, ^38^, ^55^, ^58^, ^59^, ^61^, ^82^, ^84^ frameworks went on to be used by the groups that developed them, but very few had evidence of adoption by other groups. One framework was promoted by the UK Health Technology Assessment programme as “best practice.”^38^ The most recent report‐focused framework (GRIPP2^34^) is recommended by several leading journals, though few currently make its use mandatory. Three frameworks that were developed within a particular clinical field (elderly care,^80^ rheumatology^59^, ^84^ and addiction services for marginalized groups^79^) are now used by other research teams in the same field, dissemination occurred via conferences and topic‐specific clinical research networks (personal communications from lead authors).

In only one example (Abelson et al's Public and Patient Engagement Evaluation Tool^81^), the authors, who are actively auditing use of their framework, reported widespread use of their public involvement instrument to evaluate lay involvement (personal communication from lead author). A search of the published academic literature using Google Scholar identified only rare instances of one research group describing the application of a framework developed by another group,^92^, ^99^ though we acknowledge that we may have missed other examples. Only one framework in our sample reported formal usability testing.^82^ At the time of writing, the UK PPI standards are being piloted for usability in 10 testbeds and 49 additional organizations across the UK^100^; a revised set of standards is expected to be published in 2019.

In sum, frameworks to guide patient and lay involvement in research developed in one setting do not appear to have transferred readily to other settings, except when they have been oriented to a specific clinical field and actively disseminated within that field.

Our data set also revealed a number of examples of efforts to operationalize a theoretically derived framework using some kind of practical workshop. For example, the Public Involvement Impact Assessment Framework (PiiAF)^58^ was developed using a literature review and Delphi panel to formulate draft principles^101^ and a series of facilitated workshops to address usability.^37^ Other examples of workshop formats included De Wit et al's^84^ “serious play” workshop to surface and explore researchers' willingness to share power with lay partners, and Dillon et al's^73^ facilitated workshop to finalize and operationalize their Critical Outcomes of Research Engagement (CORE) metrics for measuring the impact of lay involvement. These groups (and others in our data set^31^, ^85^, ^91^) described a positive process characterized by productive conflict which improved stakeholder engagement and partnership synergy.

### 3.8 Co‐design phase

Following our two preliminary development workshops, the three co‐design workshops involved a total of 30 participants (including people who identified primarily as patients, carers and service users, those who worked in facilitation or advocacy roles, researchers, research managers and industry representatives). Each workshop unfolded differently, with participants drawing on the resources in different ways. The workshop resources and facilitator notes (available as Appendix S2) appeared flexible and enabled the generation of widely differing frameworks designed for different purposes. All the workshops were positively evaluated; some seemed to be more successful than others (related to the maturity of the group and the quality of facilitation). None of the workshops, even those working with well‐established patient involvement groups, produced a definitive framework, which suggests that a frameworking process is likely to require a series of facilitated workshops, not a one‐off event. Additional findings from the workshop study (which is ongoing) will be reported in a subsequent paper.

## 4 DISCUSSION

### 4.1 Summary of principal findings

This study, which to our knowledge is the first attempt at a comprehensive synthesis of frameworks for supporting patient and lay involvement in health research, has produced four main findings.

First, well over 60 frameworks already exist, many though not all of which have been robustly developed using both theoretical principles and extensive patient and lay involvement.

Second, we have developed a new taxonomy of these frameworks—power‐focused, priority‐setting, study‐focused, report‐focused and partnership‐focused—based on their primary focus and intended purpose.

Third, we have ascertained that most published frameworks have been little used beyond the groups that developed them (with the exception of frameworks oriented to a particular clinical field and disseminated via networks within that field).

Finally, we have refined a provisional format and set of resources for an evidence‐based “develop your own framework” workshop to be run adaptively by researcher‐lay partnerships.

Whilst the frameworks in our data set were developed in different ways and for diverse reasons and use cases, the similarities among them were as striking as their differences. Almost all authors warned about the dangers of tokenism and tick‐box approaches; encouraged efforts to extend the diversity and representativeness of patient and lay input; emphasized that democratic values and principles must be underpinned by leadership, good governance and attention to training and practicalities; and recommended ongoing evaluation to feed into organizational learning and quality improvement. The empirical component of our study illustrated that a common set of evidence‐based resources can, when used to support facilitated design, produce different kinds of framework to suit the needs of different groups.

### 4.2 Comparison with other studies

Four previous “framework of frameworks” publications offered a taxonomy of published approaches to patient and lay involvement in health research, though each took a narrower focus than our own review. In an early non‐systematic review (written when only six of the frameworks in our sample had been published), Savory arranged previous literature broadly along two axes: focus of involvement (patient, carer, group, interested layperson, general public) and purpose of participation (“on,” “with,” “by” and “led by” lay people).^64^ Fransman explored various theoretical discourses used to analyse public engagement in research (not limited to health).^63^ Hughes and Duffy used concept analysis to consider how power‐sharing had been theorized in previous public involvement frameworks.^62^ Boivin et al^83^ summarized and critiqued evaluation tools for patient and lay involvement in research.

The emerging literature on the use of practical workshops in knowledge creation helps explain why our focus on building one's own framework appeared to be more successful than inviting groups to use off‐the‐shelf frameworks. This literature includes reviews of approaches to co‐creation of knowledge^102^ and the sociology of design,^103^ and (more specifically relevant to our empirical work) a recent theorization of “collective making.”^29^ In the last of these, Langley et al propose three domains of influence when people from different sectors come together to engage in creative play:

- influence on *participants* (creative play levels hierarchies, reduces jargon, gives voice, sparks ideas, inspires motivation, helps articulate complex ideas and concepts, and may have therapeutic value);
- influence on *knowledge* (creative play shares knowledge in many different forms, creates new knowledge, blends and synthesizes knowledge, and retains a pragmatic focus on using knowledge); and
- influence on the *process of implementation* (the intervention generated through creative play is “owned” by end‐users; the intervention incorporates research, experiential and contextual knowledge and comes with the testimony of end‐users who were involved in the making; it includes a “boundary object” in physical or visual form that acts to engage others beyond the co‐design group; and it typically includes “core” and “adaptable” elements).

The shift in our focus from identifying published frameworks to supporting local co‐design of frameworks reflects an emerging philosophical shift in the way knowledge is conceptualized: from a highly objective view of knowledge (positivism, which views knowledge as “facts” that are empirically derived and to a large extent context‐independent) and a more subjective view (interpretivism, which views knowledge as socially constructed and perspectival) to a hands‐on view of knowledge (known as performative and defined as something that is brought into being in and through human action).^102^ In other words, actively building a framework may be more effective and enduring than attempting to apply someone else's framework. Van de Ven and Johnson^104^ explain how the principles and philosophy of pragmatism (attending primarily to the practical and context‐dependent use to which the outputs of practical work will be put) can aid a performative approach to collaborative knowledge creation: “By exploiting multiple perspectives, the robust features of reality become salient and can be distinguished from those features that are merely a function of one particular view or model” (page 810).

Ours is not the first study to grapple with the tension between an academic ideal and a local, pragmatic solution. Deborah Ghate recently described an attempt to co‐produce a parenting programme that was both “evidence‐based” (ie, drawing on the research literature, which in this case was characterized by intensive interventions that were difficult to replicate and prohibitively expensive) and “home‐grown” (ie, co‐produced by local practitioners and the groups they sought to serve, taking account of contextual realities and resource constraints).^105^ Published research evidence was used to develop a sophisticated theory of change that was fed into local activities to produce what Ghate called “evidence‐supported design.”

### 4.3 Strengths and limitations

To our knowledge, this is the most comprehensive and systematic summary of patient and lay involvement frameworks yet published. The literature search was extensive and used multiple methods (including database searching, hand searching and citation tracking) to amass an extensive primary data set. Through detailed data extraction and theoretical analysis, we have produced a new taxonomy into which future studies can be classified—and which has the potential to be extended if other groups develop new approaches to exploring the field. This is also the first systematic review on this topic to have gone beyond an academic synthesis: we produced, and empirically tested, a set of resources intended for use in practical workshops, allowing different researcher‐lay partnerships to draw on them in different ways through evidence‐informed serious play.

One limitation of this review is that few primary studies were based in low‐ or middle‐income settings. A reviewer of an earlier draft of this paper suggested that not all countries or settings have a strong culture of patient involvement in research, so frameworks or framework‐building activities that implicitly assume such a culture may have limited success.

Another key limitation of this study is that the empirical component reported here was preliminary. We tested the practical resources on only three researcher‐lay partnerships, all of which were linked to the University of Oxford and did not represent the potential range of diversity of such partnerships. Whilst we believe we have demonstrated proof of concept for our “co‐design your own framework” approach, we invite other groups to explore their use of our workshop resources and facilitator guides in a wider range of target groups and settings. We have made these resources available free for download from the Health Expectations website to those using them in non‐profit initiatives.

## 5 CONCLUSION

This study has shown that numerous published frameworks for supporting and evaluating patient and public involvement in research already exist. They have different provenances, intended purposes, strengths and limitations. But being evidence‐based and theoretically informed is no guarantee that a framework will be used. A single, one‐size‐fits‐all framework may be less useful than a range of resources that can be adapted and combined in a locally generated co‐design activity.

We suggest that those who seek to develop or strengthen the patient or lay involvement in their own research use a three‐step process. First, explore the published examples described in this paper and the Appendix S1. Depending on context and intended use case, a framework may be found that is fit for purpose—perhaps with some adaptation. In the absence of such a framework, download and study the facilitator guide and evidence‐based resources and prompts, which are based on the findings of this review. Finally, work with patient collaborators and (ideally) professional facilitators to plan and deliver a series of co‐design workshops to generate a locally relevant and locally owned framework.

## CONFLICT OF INTEREST

None declared.

## ETHICS APPROVAL AND CONSENT TO PARTICIPATE

This study is part of the “Partnerships for Health, Wealth and Innovation” research stream of the Oxford Biomedical Research Centre which received ethics clearance through the University of Oxford Central University Research Ethics Committee (R51801/RE001). Written consent was obtained from patient participants.

## TRANSPARENCY DECLARATION

TG (the manuscript's guarantor) affirms that the manuscript is an honest, accurate and transparent account of the study being reported; that no important aspects of the study have been omitted; and that any discrepancies from the study as planned (and, if relevant, registered) have been explained.
