# Supplementary material for: Frameworks for supporting patient and public involvement in research: Systematic review and co‐design pilot
Source: Health Expect. 2019 Apr 22;22(4):785–801. doi: 10.1111/hex.12888 (PMC6737756; doi:10.1111/hex.12888)
Supplement: Supplementary file 1 [file HEX-22-785-s001.pdf]

Appendix S1: Additional examples of frameworks in the five categories

All diagrams are reproduced under Creative Commons licence or with permission of author.

A. EXAMPLES OF POWER-FOCUSED FRAMEWORKS

A1: Example of early power-focused framework for patient and lay involvement in research, reproduced from Oliver et al 2004[1]

		Consumers' degree of engagement			
		Consumer Control	Collaboration	Consultation	Minimal
Researchers' degree of engagement	Inviting consumer groups		Type A	Type B	
	Inviting individual consumers		Type C	Type D	
	Responding to consumer action		Type E	Type F	Type G
	Minor partner or absent	Type H			

A2: Example of power-focused framework to explore 'knowledge spaces' in researcher-lay partnerships, reproduced Gibson et al[2]

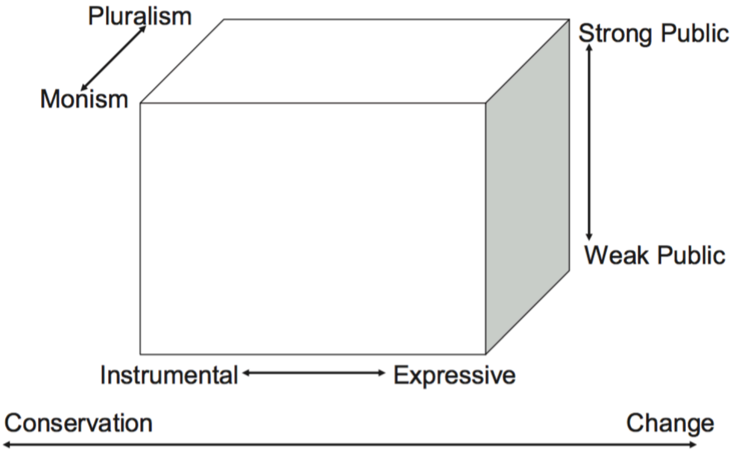

Sociological origins: weak/strong voice [Fraser], many ways/ one way to be involved [Bourdieu], organisation's concerns / public concerns [Habermas]

A3: Example of power-focused framework to identify key ethical principles for researcher-lay partnerships, reproduced from Gradinger et al[3]

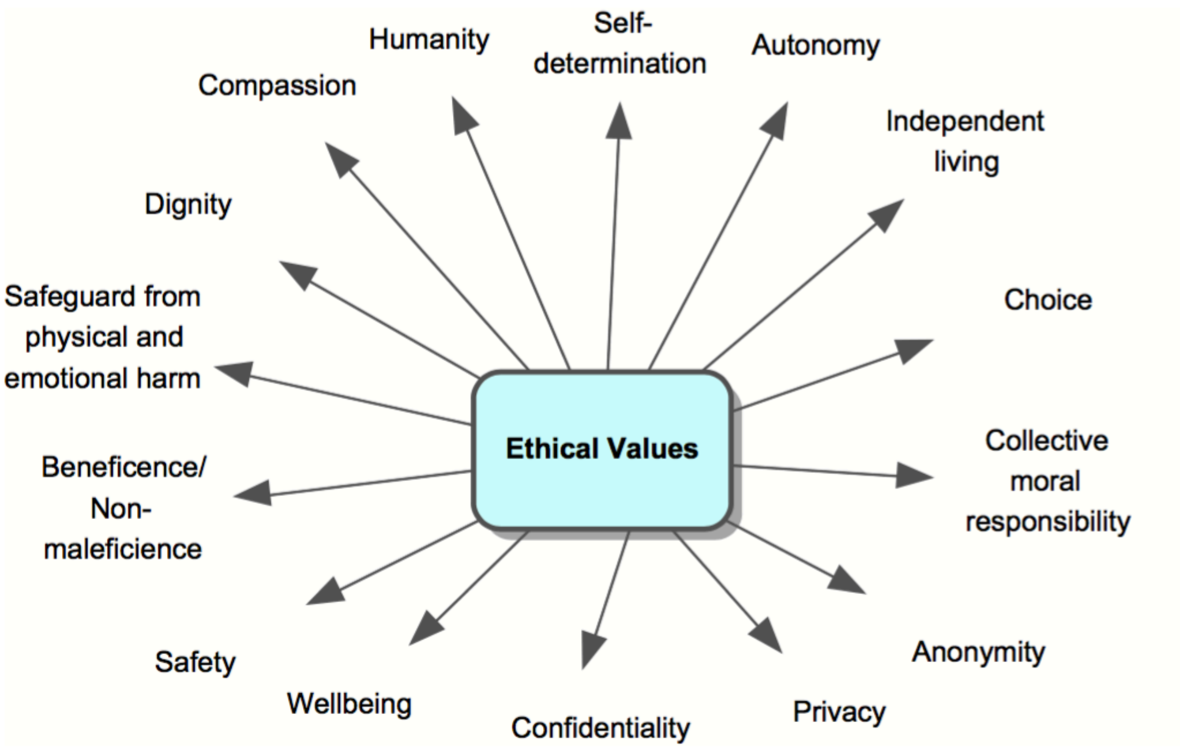

A4: Example of power-focused framework for critically exploring the level of democracy in a research partnership, reproduced from Morrow[4]

The framework is in the form of a questionnaire which patients and lay partners are invited to fill out. Each item is scored on a 5-point Likert scale (e.g. from ‘not at all’ to ‘very much’).

PART 1: PERSONAL FACTORS

1) Your ability

   To what extent do you feel you are able to…?

   a. access research resources (e.g. money, facilities, information)
   b. achieve your own goals through research
   c. make a contribution to the research
   d. make decisions about how to do the research
   e. express your views about research topics
   f. take on new research challenges

2) Your potential

   To what extent do you feel there is a potential for you to…?

   a. choose the type of role you play in research

b. bring your own ideas and values to the research
c. work in ways that suit you
d. gain status, expertise or credibility because of your involvement
e. identify and organise your research ideas and priorities

3) Your sense of being

To what extent do you feel …?

a. valued as a partner (not controlled)
f. enabled (rather than constrained)
g. empowered (rather than exploited)
h. consenting (happy to be involved) not coerced (unhappy about it)
i. it is acceptable that different people have different responsibilities and decisions to make about the research

PART 2: RESEARCH CONTEXTS

4) Research relationships

Thinking about research relationships, to what extent do you think…?

a. the researchers have the right reasons for wanting to work with you
b. there is sufficient funding to make involvement work
c. you have enough information about research involvement
d. the way the researchers work with you is supportive
e. the way the researchers communicate with you is supportive
f. the types of goals that the researchers want is what you want

5) Ways of doing research

Thinking about the research itself, to what extent do you think…?

a. there is a clear role in the research for you
b. the skills/experience needed for the role are clear to you
c. the responsibilities for the role are clear to you
d. you are aware of the legal and ethical 'rules' for doing research (e.g. confidentiality)

6) Research structures

Thinking about the research organisation, to what extent do you think your involvement is…?

a. not just part of a project, it is valued as part of the work of the organisation
b. supported by research ethics and governance systems
c. helped because of research structures (Networks, links with other studies etc)
d. noticed and recorded as part of the work of the research organisation

A5: Example of power-focused framework developed in community-based participatory research, reproduced from Belone et al[5]

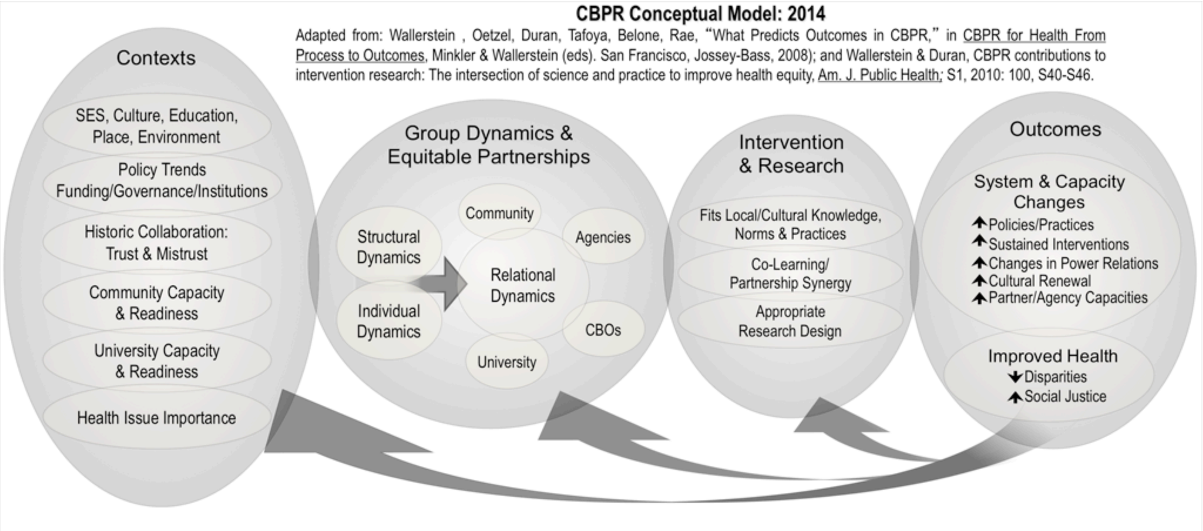

Contexts	Group Dynamics		Intervention & Research	Outcomes
• Social-Economic Status, Culture, Spirituality, Place, Education, History, Environment<br>• Policy Trends: National/Local Governance & Funding/Political Climate<br>• Role of Institutions, i.e., Education; Research (positive/negative)<br>• Historic Degree of Collaboration & Trust between Community/Academic<br>• Community: Capacity, Readiness & Experience<br>• University: Capacity, Readiness & Experience<br>• Perceived Severity of Health Issues	*Structural Dynamics*<br>• Diversity<br>• Complexity<br>• Formal Agreements<br>• Sharing Power/Resource<br>• CBPR Principles Alignment<br>• Time in partnership<br>*Individual Dynamics*<br>• Core vValues<br>• Participation Motivation<br>• Personal Relationships<br>• Cultural Identities/Humility<br>• Bridge People<br>• Personal Belief/Spirituality<br>• PI Community Reputation	*Relational Dynamics*<br>• Safety: Community Voice/ Community Language<br>• Trust<br>• Dialogue, Listening & Mutual Learning; Flexibility<br>• Leadership/Influence<br>• Power Dynamics/ Stewardship<br>• Self & Collective Reflection<br>• Participatory Decision-making & Negotiation<br>• Local knowledge Integration; Group Process<br>• Task Roles & Communication	• Intervention is Culturally Created & Centered<br>• Intervention Informed by Local Settings & Organizations<br>• Partner Shared Synergy & Trust<br>• Community Language vs. Expert Language<br>• Research & Evaluation Design Reflects Partnership Input<br>• Bidirectional Translation, Implementation & Dissemination	*CBPR System & Capacity Changes*<br>• Changes in Policies /Practices -In Universities & Communities<br>• Sustainable/Cultural-Centered interventions/Broader Reach<br>• Changed Power Relations & Empowerment -Community Voices Heard<br>• Develops Skills to Benefit Individuals & Partner Agencies<br>• Productivity Measures, i.e. Papers, Grant Applications, Grant Awards<br>• Cultural Revitalization & Renewal<br>*Health Outcomes*<br>• Transformed Social& Economic Conditions<br>• Reduced Health Disparities

B. EXAMPLE OF PRIORITY-SETTING FRAMEWORKS

B1: Example of priority-setting framework, reproduced from Viergever et al[6]

**1. Context**
Decide which contextual factors underpin the process: What resources are available for the exercise? What is the focus of the exercise (i.e. what is the exercise about and who is it for)? What are the underlying values or principles? What is the health, research and political environment in which the process will take place?
**2. Use of a comprehensive approach**
Decide if use of a comprehensive approach is appropriate, or if development of own methods is the preferred choice. These approaches provide structured, detailed, step-by-step guidance for health research priority setting processes from beginning to end.
**3. Inclusiveness**
Decide who should be involved in setting the health research priorities and why. Is there appropriate representation of expertises and balanced gender and regional participation? Have important health sectors and other constituencies been included?
**4. Information gathering**
Choose what information should be gathered to inform the exercise, such as literature reviews, collection of technical data (e.g. burden of disease or cost-effectiveness data), assessment of broader stakeholder views, reviews or impact analyses of previous priority setting exercises or exercises from other geographical levels.
**5. Planning for implementation**
Establish plans for translation of the priorities to actual research (via policies and funding) as a priority at the beginning of the process. Who will implement the research priorities? And how?
**Deciding on priorities**
**6. Criteria**
Select relevant criteria to focus discussion around setting priorities.
**7. Methods for deciding on priorities**
Choose a method for deciding on priorities. Decide whether to use a consensus based approach or a metrics based approach (pooling individual rankings), or a combination.
**After priorities have been set**
**8. Evaluation**
Define when and how evaluation of the established priorities and the priority setting process will take place. Health research priority setting should not be a one-time exercise!
**9. Transparency**
Write a clear report that discusses the approach used: Who set the priorities? How exactly were the priorities set?

C. EXAMPLES OF STUDY-FOCUSED FRAMEWORKS

C1: Example of study-focused framework: Patient and Service User Involvement framework from PCORI (Patient Centered Outcomes Research Institute), reproduced from Shippee et al[7]

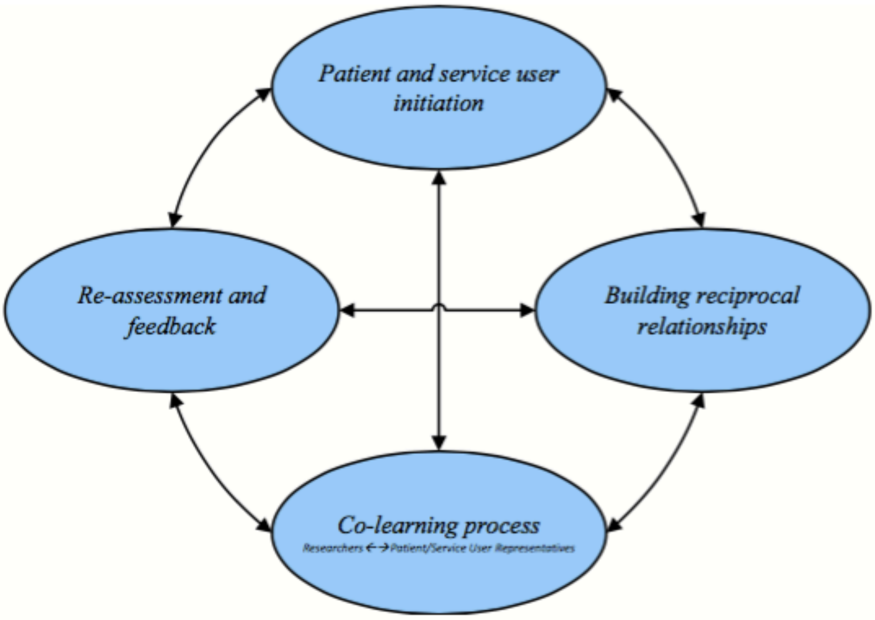

Figure 2 Components of patient and public involvement in research.

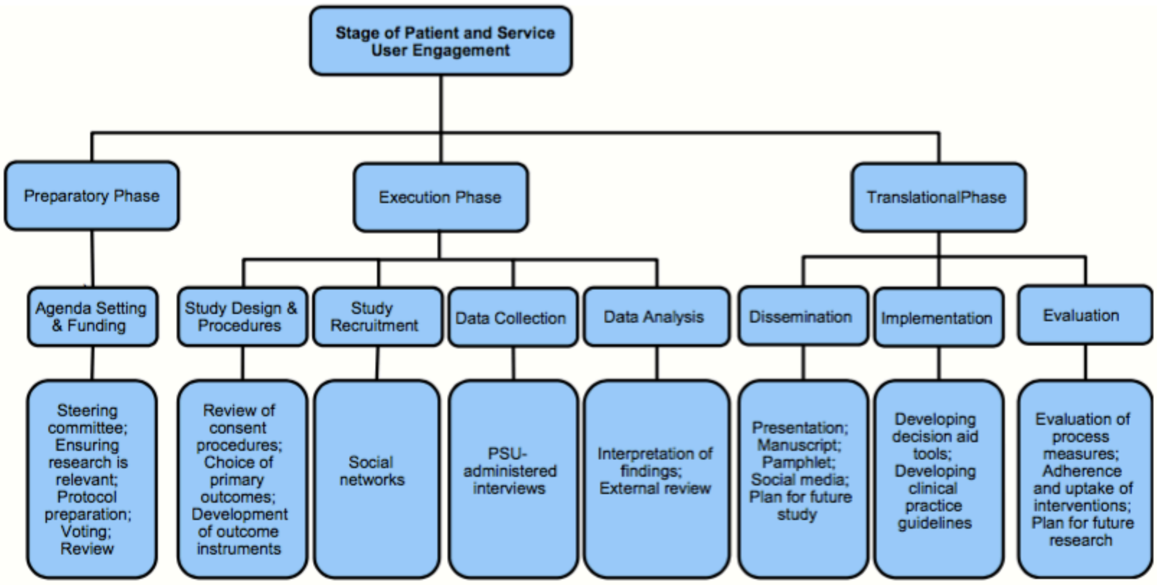

Figure 3 Phases and stages of patient and service user engagement in research.

C2: Example of study-focused framework that includes measurement of the impact of patient and lay involvement, reproduced with permission from Dillon et al[8]

See also Figure 5 in main paper.

Table 2. Critical Outcomes of Research Engagement (COREs) Definitions, Related Measures

CORE	Measures
**Patient-centered** – Research centered around patients' values, beliefs and experiences. Anticipates participant issues, respects and reflects patient experience and validates patient partner input/contributions. Engages patients in a respectful, culturally appropriate and condition/disease-sensitive manner.	1. How did patient partners influence each stage of research and critical research tasks?<br>2. Changes in specific outcomes (response rates, retention, etc.) relative to other similar studies without patient partners.
**Meaningful** – Research/methods/outcomes are reflective of and relevant/meaningful to the community as well as impactful (as perceived by community and all stakeholders).	1. Are potential outcomes, comparators and content meaningful to patient community?
**Team collaboration ("teamness")** – Having an appropriate, collaborative team that includes a variety of researcher and patient expertise and experiences. Capacity building includes patient partners gaining skills and knowledge through experience and/or training.	1. What is the patient partner's comfort level during discussions?<br>2. What is the patient partner's comfort level with written materials?<br>3. Do all team members trust and respect each other?
**Understandable** – Use of engaging common language that includes specific and measurable questions, agreed on by both researchers and partners. All research materials are understandable to participants and patient partners.	1. Are study materials understandable and written in a common/plain language?<br>2. Evaluate the reading level of research documents.<br>3. How were patient partners trained and supported in their research work?<br>4. Did researchers present data to patient partners in an accessible, understandable way?
**Rigorous** – Research is generalizable, reliable and validated by the patient partners and reflects the diverse participants in an ethical, unbiased and timely fashion. Honest/accurate results.	1. Did the research team use "realistic continuous improvement" methods to maintain scientific rigor while also incorporating partner suggestions?<br>2. Checklist of stages/discrete decisions for which patient partners were consulted and had meaningful influence.<br>3. Did patient partners propose any changes that were not made? If so, explain why.
**Integrity/Adaptable** – Research maintains balance between process improvement and study goals. Willingness to change study design through "realistic continuous improvement."	1. Is the research question clear and understood by everyone?<br>2. To what extent do patient partners contribute to creating a fair, ethically sound research study (document changes in study design, methods, materials, etc.)?
**Legitimate** – Findings are considered legitimate and trusted by relevant communities, increasing likelihood results will be translated/adopted due to buy-in.	1. To what extent were partners involved in each stage of research? How were their insights incorporated?<br>2. To what degree was the sample or study population diverse and representative/unbiased?
**Feasible** – Identify/address assumptions to make goals and methods realistic.	1. Are research goals and methods realistic and feasible?
**Ethical and transparent** – Patient partners ensure transparency, fairness, truly informed consent and participation, and continuously check assumptions of research team members.	1. Are all methods and materials patient-friendly?<br>2. How does the study design, including data collection methods, accommodate and show respect for participant diversity?<br>3. Is data/privacy protection more patient-centered and/or changed?
**Timely** – Timely analysis and reporting; iterative data sharing with patient partners.	1. Is conduct of research and sharing information with patient partners timely?
**Sustainable** – Research has long-term value; patient partner relationships are maintained over time.	1. What different mediums were used to disseminate findings, and where were results shared?<br>2. Were results translated or adopted outside the research study?<br>3. What role did patient partners play in dissemination?

C3: Example of study-focused framework: realist model of context-mechanism-outcome configurations in efforts to improve patient and public involvement in a research study, reproduced from Evans et al[9]

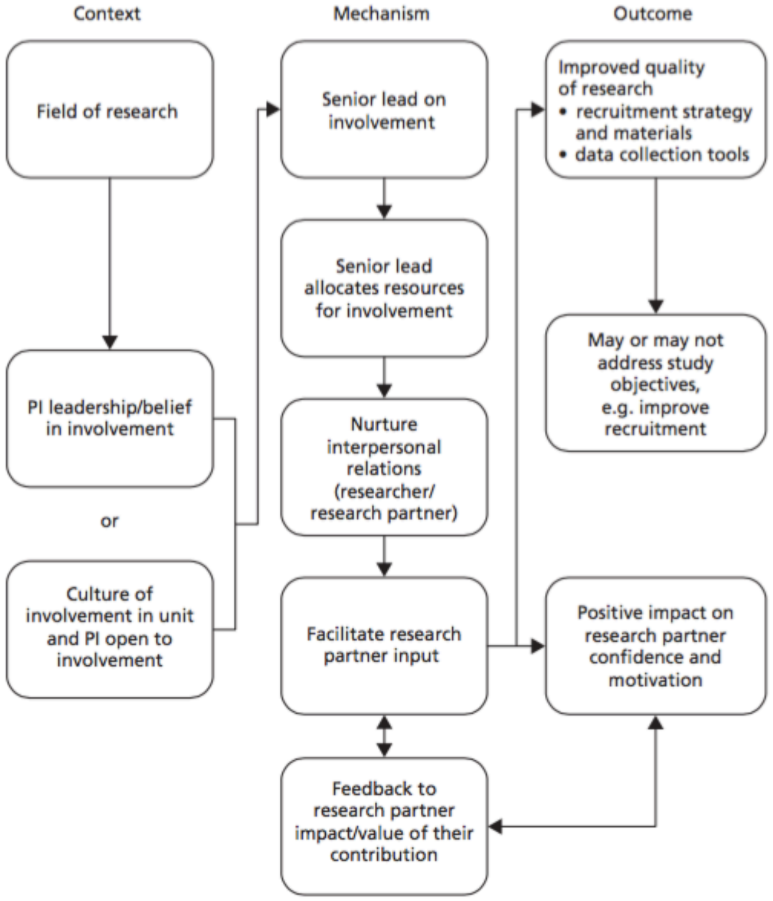

FIGURE 1 Context, mechanism and outcome pathway.

D. EXAMPLES OF REPORT-FOCUSED FRAMEWORKS

D1: Long form of GRIPP2, reproduced from Staniszewska et al[10]

Section and topic	Item	Reported on page No
Section 1: Abstract of paper		
1a: Aim	Report the aim of the study	
1b: Methods	Describe the methods used by which patients and the public were involved	
1c: Results	Report the impacts and outcomes of PPI in the study	
1d:Conclusions	Summarise the main conclusions of the study	
1e: Keywords	Include PPI, “patient and public involvement,” or alternative terms as keywords	
Section 2: Background to paper		
2a: Definition	Report the definition of PPI used in the study and how it links to comparable studies	
2b: Theoretical underpinnings	Report the theoretical rationale and any theoretical influences relating to PPI in the study	
2c: Concepts and theory development	Report any conceptual models or influences used in the study	
Section 3: Aims of paper		
3: Aim	Report the aim of the study	
Section 4: Methods of paper		
4a: Design	Provide a clear description of methods by which patients and the public were involved	
4b: People involved	Provide a description of patients, carers, and the public involved with the PPI activity in the study	
4c: Stages of involvement	Report on how PPI is used at different stages of the study	
4d: Level or nature of involvement	Report the level or nature of PPI used at various stages of the study	
Section 5: Capture or measurement of PPI impact		
5a: Qualitative evidence of impact	If applicable, report the methods used to qualitatively explore the impact of PPI in the study	
5b: Quantitative evidence of impact	If applicable, report the methods used to quantitatively measure or assess the impact of PPI	
5c: Robustness of measure	If applicable, report the rigour of the method used to capture or measure the impact of PPI	
Section 6: Economic assessment		
6: Economic assessment	If applicable, report the method used for an economic assessment of PPI	
Section 7: Study results		

Section and topic	Item	Reported on page No
7a: Outcomes of PPI	Report the results of PPI in the study, including both positive and negative outcomes	
7b: Impacts of PPI	Report the positive and negative impacts that PPI has had on the research, the individuals involved (including patients and researchers), and wider impacts	
7c: Context of PPI	Report the influence of any contextual factors that enabled or hindered the process or impact of PPI	
7d: Process of PPI	Report the influence of any process factors, that enabled or hindered the impact of PPI	
7ei: Theory development	Report any conceptual or theoretical development in PPI that have emerged	
7eii: Theory development	Report evaluation of theoretical models, if any	
7f: Measurement	If applicable, report all aspects of instrument development and testing (eg, validity, reliability, feasibility, acceptability, responsiveness, interpretability, appropriateness, precision)	
7g: Economic assessment	Report any information on the costs or benefit of PPI	
Section 8: Discussion and conclusions		
8a: Outcomes	Comment on how PPI influenced the study overall. Describe positive and negative effects	
8b: Impacts	Comment on the different impacts of PPI identified in this study and how they contribute to new knowledge	
8c: Definition	Comment on the definition of PPI used (reported in the Background section) and whether or not you would suggest any changes	
8d: Theoretical underpinnings	Comment on any way your study adds to the theoretical development of PPI	
8e: Context	Comment on how context factors influenced PPI in the study	
8f: Process	Comment on how process factors influenced PPI in the study	
8g: Measurement and capture of PPI impact	If applicable, comment on how well PPI impact was evaluated or measured in the study	
8h: Economic assessment	If applicable, discuss any aspects of the economic cost or benefit of PPI, particularly any suggestions for future economic modelling.	
8i: Reflections/critical perspective	Comment critically on the study, reflecting on the things that went well and those that did not, so that others can learn from this study	

PPI=patient and public involvement

D2: Example of report-focused framework for reporting patient and lay involvement in systematic reviews, reproduced from Pollock 2018[11]

Framework Constructs	***Categories***		***Key / Icon***
**Who is involved?**	Patients, carers and / or their families		
	Patients, carers and / or their families + other stakeholders		
	Other stakeholders only		
**How are people recruited?**	Open	Fixed	Fixed
		Flexible	Flexible
	Closed	Invitation	Invite
		Existing group	Group
		Purposive sampling	Sample
	Other / Unclear		?
**What happened?** *Approach?*	One-time		x1
	Continuous		
	Combined (i.e. both one-time and continuous)		
**What happened?** *Methods?*	Direct interaction		
	No direct interaction		
**Stage & Level?**	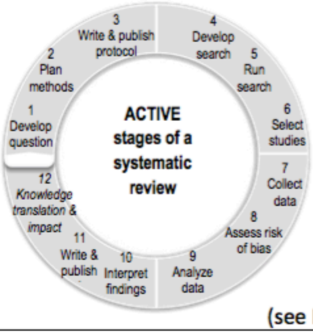 (see Figure 2)	Leading	Lead
		Controlling	Control
		Influencing	Influen
		Contibuting	Contrib
		Receiving	Receive
	Top & tail approach?		1 12

FIGURE 1: The ACTIVE framework of involvement in a systematic review

Under Review

E. EXAMPLES OF PARTNERSHIP-FOCUSED FRAMEWORKS

E1: Partnership principles developed by Boote et al[12]

Principle	Indicator
1. The roles of consumers are agreed between researchers and consumers	The roles of consumers in the research were documented
2. Researchers budget appropriately for the costs of consumer involvement	Researchers applied for funding to involve consumers<br>Consumers were reimbursed for their travel costs<br>Consumers were reimbursed for their indirect costs e.g. carer costs
3. Researchers respect the differing skills, knowledge and experience of consumers	The contribution of consumers' skills, knowledge and experience was included in research reports and papers
4. Consumers are offered training and personal support, to enable them to be involved in research	Consumers' training needs for research involvement were agreed between consumers and researchers<br>Consumers had access to relevant training<br>Mentors were available to provide personal and technical support to consumers
5. Researchers ensure that they have the necessary skills to involve consumers in the research process	Researchers ensured that their own training needs were met in elation to involving consumers in research
6. Consumers are involved in decisions about how participants are both recruited and kept informed about the progress of the research	Consumers gave advice to researchers about how to recruit participants<br>Consumers gave advice to researchers about how to keep participants informed
7. Consumer involvement is described in research reports	The involvement of consumers is acknowledged in research reports and publications<br>Details are given in the research reports of how consumers were involved
8. Research findings are available to consumers in formats and language they can easily understand	Research findings were disseminated to consumers in appropriate formats e.g. large print, translations, audio, Braille<br>The distribution of research findings to consumers was in appropriate formats<br>Consumers gave their advice on the choice of methods used for dissemination

E2: Example of partnership-focused framework derived from a Delphi exercise, reproduced from Baines and Regan de Bere[13]

PPI Principles rank ordered by expert respondents
1. Share information, experiences, knowledge and power
2. Listen, assess and respond to information shared. Regularly update people involved. Do not collect information and then ignore it as this is disrespectful and tokenistic. Act on information shared and offer clear explanations as to why suggested changes have not been acted on.
3. Work in equal partnerships built on mutual trust, respect and transparency.
4. Communicate and inform regularly, clearly and inclusively. Do not rely on one method of communication; this is unlikely to be suitable for all those involved – be creative.
5. Support and prepare everyone involved before, during and after any working together initiative. This includes offering relevant training, information, practical, emotional and financial support.
6. Acknowledge, reward and value everyone involved. Celebrate good practice.
7. Accommodate individual and collective needs to ensure inclusivity.
8. Commit to working together on a personal, organisational and long term basis.
9. Be proactive. Go out to relevant communities and get involved. Do not expect people to come to you.
10. Resource and invest. Effective working together takes time, money and resources. Be prepared to invest time and effort; it will nearly always take longer than you think.
11. Empower all members involved. Ensure information, resources and skills are shared so everyone can contribute to decision-making processes.
12. Tailor your approach, materials, training and evaluations provided to match your aim, purpose and local context.
13. Evaluate throughout your working together initiative to identify best practice and areas that can be improved.

E3: Example of partnership-focused framework (FIRST) developed in a single clinical field (rheumatology) and disseminated through conferences and research networks in that field. Later paper[14] reports additions and clarifications to an earlier version[15]

Box 1 The original FIRST model[41] and the clarification and additions

Original First model	Clarification and additions
**Facilitate**	
Facilitating the involvement of partners in team meetings (e.g. by providing reimbursement and working in pairs). Inclusion at an early stage, when partners can fully contribute to the research questions and the desired methods and outcomes, is preferable. The role of the principal investigator (PI) is a key	Facilitate refers to creating practical conditions and eliminating barriers for structural collaboration. It deals with procedural, environmental and physical factors. The PI is responsible for facilitating inclusive conditions for participative research, among which adequate instruction and support of the actual researcher are important
**Identify**	
Identify partners	*Identify partners*
It is recommended that suitable partners are identified through the clinic or through patient organizations. Partners require in-depth experience with health issues, an ability to review and discuss information, and the confidence to step out of the 'patient' role	Identifying partners through professionals in the clinic in which the partners will be working proved to be the preferred method of recruitment<br>Clear guidance for researchers should be provided regarding minimum selection criteria
Identify projects	*Identify projects*
Projects addressing clinical interventions, outcomes or service delivery issues could benefit most from partner involvement.	More detailed criteria need to be developed for identifying projects that are likely to benefit from patient involvement
Identify tasks	*Identify tasks*
The term 'roles' refers to research tasks that partners fulfil, such as reviewing draft protocols and questionnaires, analysing qualitative data, interpreting results and giving presentations. A job description is helpful	Separating 'roles' from 'tasks' bring clarity into the dialogue between partners and professionals. Partners can have different roles representing different levels of involvement. Tasks refer to practical activities that partners can do to contribute in subsequent phases of the research process. Roles as well as tasks need to be regularly evaluated
	Identify professionals
	In the context of implementing a network of partners, it is useful to add the concept of 'identifying professionals' to the model. Relevant criteria include motivation and the ability to accept partners as collaborating members of the research team
**Respect**	
For a successful partnership, mutual respect is a prerequisite. Respect is associated with confidentiality and acknowledgement of the contribution of the partner.	Recurrent reflection on the quality of the collaboration to discuss questions about communication, sharing power, feeling part of the team and feeling rewarded for contributions is important. All participants need to be aware of the dynamics of establishing new partnerships by recognizing that individual learning curves are valuable outcomes of the collaboration
**Support**	
Support is defined as all actions taken to help partners to work and communicate in a successful partnership, for example, by creating peer support or organizing a work place at the institute	Support refers to individual encouragement, communication and personal empowerment<br>Support should also be offered to professionals. Skills for and attitudes towards creating equal partnerships do not come automatically. Professionals require support tailored to their personal needs and competences. This kind of support might be organized under the supervision of the PI
**Training**	
Training is considered essential for partners. During training, the focus should lie on basic understanding of the research process and of measuring outcomes	Partners should be explained in advance about the limitations set by ethics committees, national law or scientific rigour. For example, partners should understand that validated questionnaires cannot be adjusted easily<br>Professionals should be provided with information about the principles of participative research, how to include partners in their projects, examples of the added value of experiential knowledge and practical dos and don'ts related to communication

E4: Example of partnership-focused framework ('Ethical Principles of Partnership') developed by an EU-funded collaboration led by patient organisations[16]

Mutual Respect
Central to all partnerships between Patient Organisations and other stakeholders in clinical research is that all parties in the partnership act in accordance with the principles of mutual respect. This means that all partners' competence, capabilities, and limitations shall be taken into account and respected. Sponsors and investigators should know, understand and respect the environment and constraints within which patient groups work.

Trust
Partnership between Patient Organisations, industry, clinicians and all other stakeholders involved must be based on mutual trust. This trust should stem from openness about motives and the confidence that all parties are working towards a common goal, even if the approaches are made from different perspectives.

Integrity and Credibility
The integrity, credibility and independence of all involved partners, as well as the constraints and obligations under which all stakeholders operate should be respected at all times when negotiating the terms of any partnership.

Reliability
In order to ensure a fruitful partnership, it is important that terms of agreement are set up at the beginning of the collaboration and that these are adhered to throughout the entire research process. We recommend periodic reviews that will evaluate the progress based on the goals and objectives. It is also advisable to agree up front how conflicts will be resolved and what are the terms for the readjustment or termination of partnership.

Accountability
It is preferable to outline in the early stages of the partnership development how each party will be held accountable for its respective input and the outcomes achieved through the collaboration. The ways in which parties will report back to their members, colleagues, partners and general public should be determined at the start.

Acknowledgement
Agreement as to how each party will be acknowledged for its contribution should be reached before the start of any collaboration. Ownership and intellectual property rights of materials produced in the collaboration should be agreed upon, taking into consideration that Patient Organisations do not necessarily have access to legal advisors and therefore need the terms to be set out in clear and simple language. Endorsement should also be discussed along with the terms of usage of the name, brand and/or logo of all parties including the names of the representatives.

Transparency
Transparency means ensuring that all parties are clear about each partner's role and responsibility within the partnership. Resources contributed by each party should be used appropriately and any other collaboration that might influence the partnership should be disclosed.

Sustainability
Collaborations should strive for a sustainable benefit for patients, rather than aiming for short-term goals or competitive advantage. This sustainability can be achieved by ensuring that the demands on administrative efforts from Patient Organisations be minimised so that the limited resources can be utilised in other areas. Results of a trial should also aim to be made public after the conclusion of the collaboration to create value for the whole community rather than just competitive advantage for the partners.

E5: Example of partnership-focused framework ('Strategy for Patient-Oriented Research') produced by a national research funder (Canadian Institute of Health Research)[17]

http://www.cihr-irsc.gc.ca/e/45851.html

Guiding principles: 1) Inclusiveness; 2) Support; 3) Mutual respect; 4) Co-build.

Core areas for engagement: 1) Governance and decision-making; 2) Capacity-building; 3) Tools and resources; 4) Evaluation.

Successful patient engagement includes: 1) Inclusive mechanisms and processes; 2) Multi-way capacity building; 3) Multi-way communication and collaboration; 4) Experiential knowledge valued as evidence; 5) Patient-informed and directed research; 6) A shared sense of purpose.

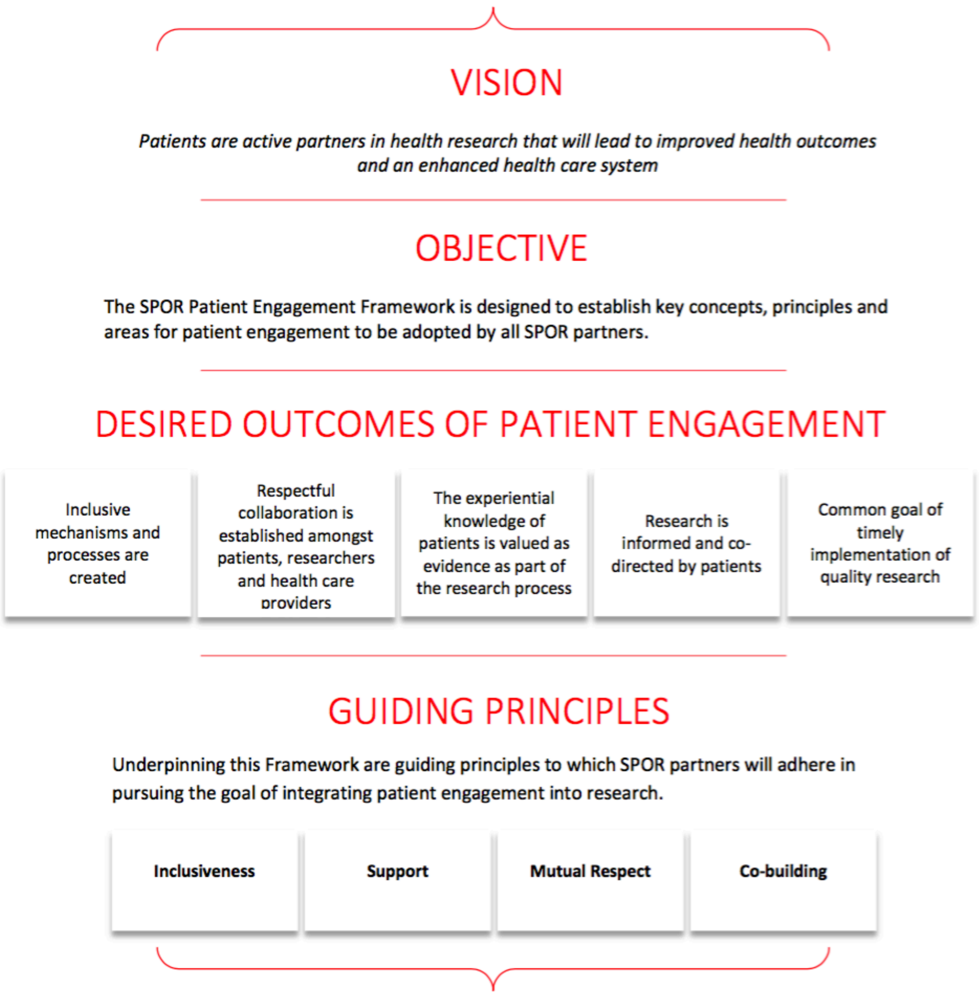

E6: Example of partnership-focused framework (patient and public engagement evaluation tool) that was tested for usability and refined[18]

Table 3 Final list of principles and prioritized outcomes

PPE Principle	Prioritized outcomes
Integrity of design and process	PPE participants represent the diverse range of views of those most affected by the decision<br>Participants are provided with access to supports to enable participation, for example:<br>PPE meeting-related expenses are covered<br>PPE activity location/amenities/time of day/day of event provide a comfortable, non-threatening environment<br>Relevant information, produced at an appropriate education level, is shared with participants<br>Clear, two-way communication exists between organizers and participants:<br>The process and objectives are clearly communicated<br>Participants understand how their input will be used<br>Input from all participants is gathered through the process<br>The outputs of the PPE activity process are reported to participants, including how their input will be used in the decision
Influence and impact	PPE informs planning/decision making<br>PPE improves participant knowledge of:<br>PPE issue<br>Organization<br>Health system<br>Public/patient perspectives (if tailored to staff)<br>Other topic of focus<br>PPE leads to increased confidence/trust in:<br>Providers<br>PPE staff<br>Organization as a whole<br>Health system<br>Personal competency (e.g., in diabetes management)
Participatory culture	The organization promotes and supports ongoing quality public engagement in strategic planning, policy and service delivery by:<br>Embedding PPE values and principles in the organization's philosophy and structure<br>Organizational leaders and managers have received training in PPE<br>PPE practice is being implemented in service and policy work<br>PPE is part of standardized business and planning processes
Collaboration and common purpose	The organization and other external community partners plan and work together to address the concerns of the people they serve

E7: Example of partnership-focused framework (patient engagement quality guidance) developed for planning, assessment and gap analysis in medicines development, reproduced from PFMD[19]

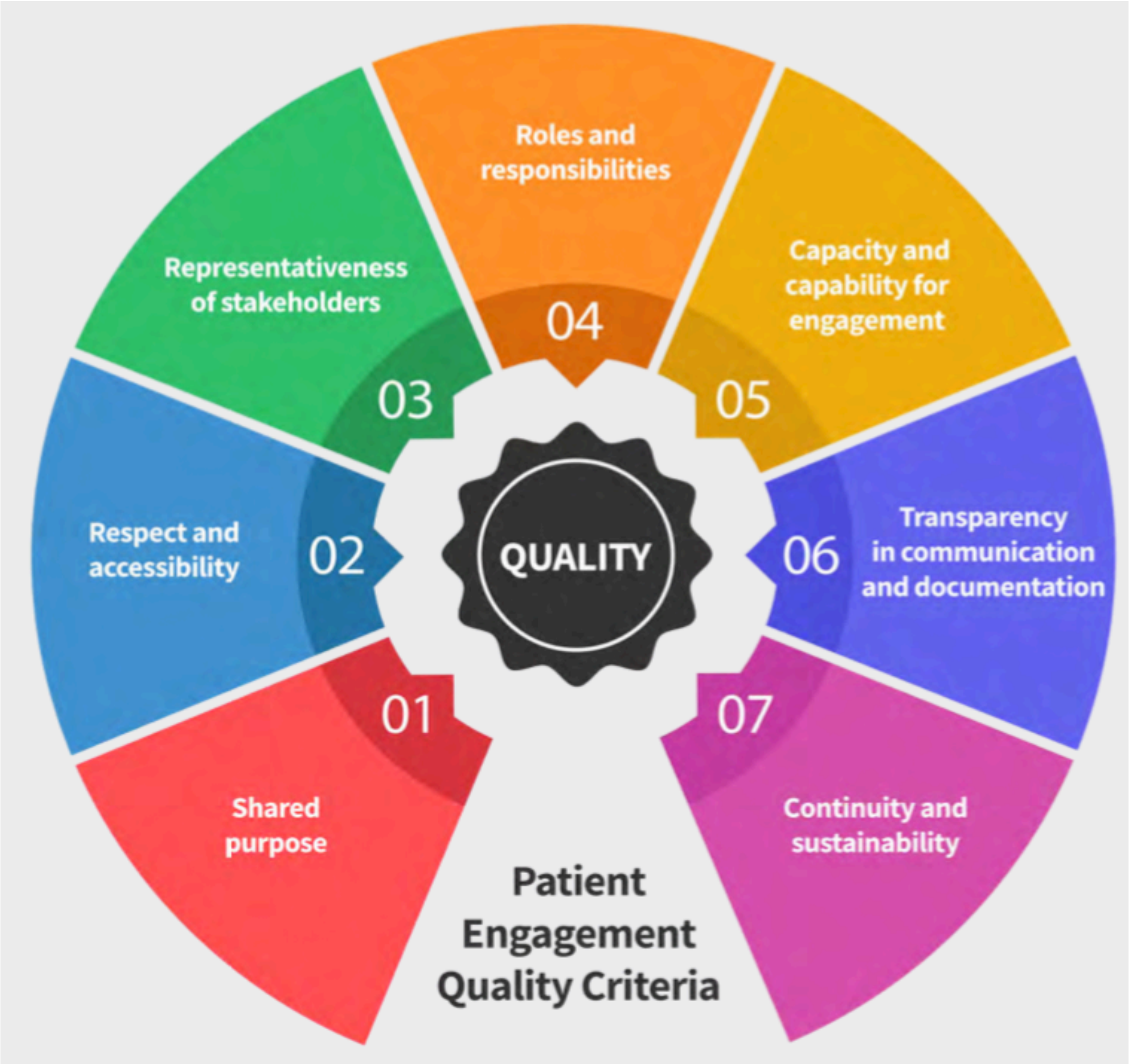

E8: Example of partnership-focused framework: ADVANCE (Accelerating Data Value Across a National Community Health Center Network) for involving patients and lay people in research on routinely collected electronic data in their health system, reproduced from Warren et al[20]

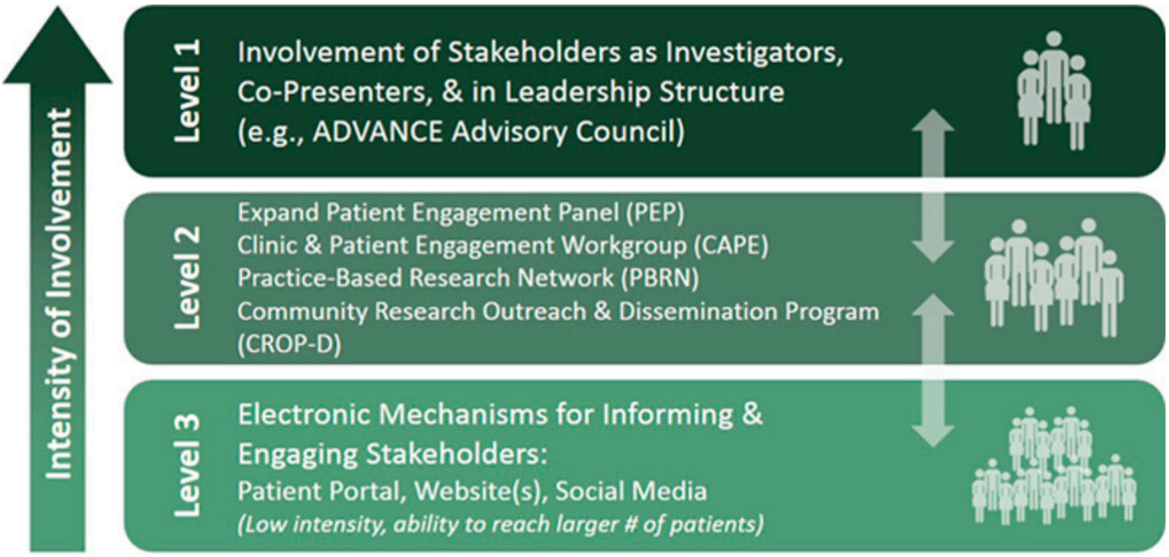

1. Oliver S, Clarke-Jones L, Rees R, et al. Involving consumers in research and development agenda setting for the NHS: developing an evidence-based approach. 2004;8(1-148)
2. Gibson A, Britten N, Lynch J. Theoretical directions for an emancipatory concept of patient and public involvement. *Health:* 2012;16(5):531-47.
3. Gradinger F, Britten N, Wyatt K, et al. Values associated with public involvement in health and social care research: a narrative review. *Health Expectations* 2015;18(5):661-75.
4. Morrow E, Ross F, Grocott P, et al. A model and measure for quality service user involvement in health research. *International Journal of Consumer Studies* 2010;34(5):532-39.
5. Belone L, Lucero JE, Duran B, et al. Community-based participatory research conceptual model: Community partner consultation and face validity. *Qualitative Health Research* 2016;26(1):117-35.
6. Viergever RF, Olifson S, Ghaffar A, et al. A checklist for health research priority setting: nine common themes of good practice. *Health research policy and systems* 2010;8(1):36.
7. Shippee ND, Domecq Garces JP, Prutsky Lopez GJ, et al. Patient and service user engagement in research: a systematic review and synthesized framework. *Health Expectations* 2015;18(5):1151-66.
8. Dillon EC, Tuzzio L, Madrid S, et al. Measuring the Impact of Patient-Engaged Research: How a Methods Workshop Identified Critical Outcomes of Research Engagement. *Journal of Patient-Centered Research and Reviews* 2017;4(4):237-46.
9. Evans D, Coad J, Cottrell K, et al. Public involvement in research: assessing impact through a realist evaluation. *Health Serv Deliv Res* 2014;2:36.
10. Staniszewska S, Brett J, Simera I, et al. GRIPP2 reporting checklists: tools to improve reporting of patient and public involvement in research. *Research Involvement and Engagement* 2017;3(1):13.
11. Pollock A, Campbell PE, C. S, et al. Development of the ACTIVE framework to describe stakeholder involvement in systematic reviews. *Journal of Health Services research and Policy* 2018;in press (epub ahead of print)
12. Boote J, Barber R, Cooper C. Principles and indicators of successful consumer involvement in NHS research: results of a Delphi study and subgroup analysis. *Health Policy* 2006;75(3):280-97.
13. Baines RL, Regan de Bere S. Optimizing patient and public involvement (PPI): Identifying its "essential" and "desirable" principles using a systematic review and modified Delphi methodology. *Health Expectations* 2017
14. De Wit M, Elberse JE, Broerse JE, et al. Do not forget the professional–the value of the FIRST model for guiding the structural involvement of patients in rheumatology research. *Health Expectations* 2015;18(4):489-503.
15. Hewlett S, Wit Md, Richards P, et al. Patients and professionals as research partners: challenges, practicalities, and benefits. *Arthritis Care & Research: Official Journal of the American College of Rheumatology* 2006;55(4):676-80.
16. PatientPartner Project. Ethical principles of partnership between researchers and the public. Accessed 11.10.17 on http://www.patientpartner-europe.eu Brussels: European Commission 2011.

17. Canadian Institutes of Health Research. Strategy for Patient-Oriented Research: Patient Engagement Framework. Accessed at http://www.cihr-irsc.gc.ca/e/48413.html on 28.8.17. Ontario: CIHR 2017.
18. Abelson J, Wagner F, DeJean D, et al. Public and Patient Involvement in Health Technology Assessment: A Framework for Action. *Int J Technol Assess Health Care* 2016;32(4):256-64. doi: 10.1017/S0266462316000362 [published Online First: 2016/09/28]
19. Patient Focused Medicines Development. Patient Engagement Quality Guidance. Accessed 12.8.18 on http://patientfocusedmedicine.org/peqg/patient-engagement-quality-guidance.pdf. Brussels, Belgium: PFMD 2018.
20. Warren NT, Gaudino JA, Jr., Likumahuwa-Ackman S, et al. Building Meaningful Patient Engagement in Research: Case Study From ADVANCE Clinical Data Research Network. *Medical care* 2018;56 Suppl 10 Suppl 1:S58-s63. doi: 10.1097/mlr.0000000000000791 [published Online First: 2018/08/04]
