# Supplementary material for: Frameworks for supporting patient and public involvement in research: Systematic review and co‐design pilot
Source: Health Expect. 2019 Apr 22;22(4):785–801. doi: 10.1111/hex.12888 (PMC6737756; doi:10.1111/hex.12888)
Supplement: Supplementary file 2 [file HEX-22-785-s002.pdf]

Appendix S2: Facilitator notes for PPI build-your-own-framework workshop

These notes are intended to support a facilitated workshop between researchers, clinicians (possibly) and patients, carers and lay people (definitely). The *goal* of the workshop is to develop a **framework** that will support the delivery of patient and public involvement in your research locally, and consider how that involvement will be supported and evaluated. As you plan, give some thought to how participants will travel, and whether and how they will be reimbursed for their input.

Workshop objectives [Please amend these to suit. For example…]

- Share what we are already doing well with PPI in our research and any areas for improvement
- Engage in a facilitated discussion about how we would like PPI to work in our research: principles and practicalities
- Based on our discussion, agree and construct a framework for how we will carry out and evaluate PPI
- Start planning how we will work together to implement the framework

Participants

[Insert details about who is attending and their role – for example…]
16 people in total, comprising

- vv patients or carers of people with ….
- ww members of the lay public
- xx clinicians
- yy researchers
- zz research nurses
- nn research managers
- qq PPI advisor to the research group

The workshop will last about 3 hours including informal arrival time and a break, roughly as follows:

ARRIVAL, REGISTRATION AND TEA/COFFEE 30 minutes

Set an arrival time half an hour before you plan to actually start, and make some refreshments available. This will allow people to arrive, get a drink and chat informally before the formal work of the session begins. It will also create a space to accomodate late arrivals (e.g. those dependent on public transport). Note: when planning the start time, try to avoid peak rush-hour times for travel.

INTRODUCTIONS, ORIENTATION, GROUND RULES 20 minutes

Welcome to the workshop by Facilitator(s).

Get people to **introduce** themselves. Use a levelling technique such as the no-cheeses rule ("say who you are but not how important you are"). Ask participants to keep introductions brief. You might like to use a simple (no cringe) ice-breaker such as "tell us on interesting thing about yourself". Informal name badges may help (perhaps get people to make their own).

Discuss **objectives (see above)**. For example, we are here today to:

- Learn and celebrate what we're already doing well in PPI
- Highlight where we could strengthen and support our PPI activities
- Plan how we would like to work together, and produce a visual representation of our ideas

Brainstorm **ground rules** for the session. Write these down (e.g. on a flip chart). These might include respecting everyone's view, letting everyone have a say, no jargon / feeling free to ask for jargon busting and so on. (Keep a Jargon Busting sheet of A1 paper on the wall and add to it whenever someone asks for a word/abbreviation to be explained.) Say that we can add more ground rules if new issues come up later.

Divide into **groups**
People work best in groups of three to six people. Each group should include a mix of patients and carers, researchers and clinicians. If there are five or fewer people, they can all work as one group.

GROUP TASK 1 45 minutes

Building the framework

This task can be done with foam shapes (try Amazon.com) or if preferred, pieces of felt or flip-chart paper – anything you can write on and move around. People can write directly on the foam or on sticky labels. Try to get everyone to join in – either by contributing ideas or by writing and arranging the ideas to make a map.

1. Start by putting the main **building blocks or themes** in place. Depending on the size of the small groups each group should focus on building frameworks around one or two main themes.

   Take a large piece of foam (or similar), and label it in big letters with one theme.

   Here are some examples:

   UNDERPINNING VALUES

   PEOPLE AND RELATIONSHIPS

SET-UP AND PRACTICALITIES

WAYS PATIENTS CAN CONTRIBUTE

If participants want to put other theme titles as well as or instead of these, that's fine.

2. Give out laminated instruction sheets, one for each of the themes above, and encourage people to work creatively with the 'foam play' to create a visual map of elements relevant to each theme. If the workshop is large, it is likely that different groups will work on different parts of the map. For example with 16 people in total, you could have four groups of four, each working on one theme.

   It's important for participants to think about how they will put their ideas into practice, rather than simply coming up with abstract ideas.

For example:

a. If people come up with **respect** under the 'Values' theme, they need to think about what that will actually entail: What behaviours come across as (dis)respectful? What will each member of the team commit to doing / not doing?
b. If people come up with **keep PPI contributors updated** under 'People and relationships', they need to think about *who* is responsible for updating people, *what* information contributors need, *how often,* etc.

Here's an example (but it doesn't have to look like this):

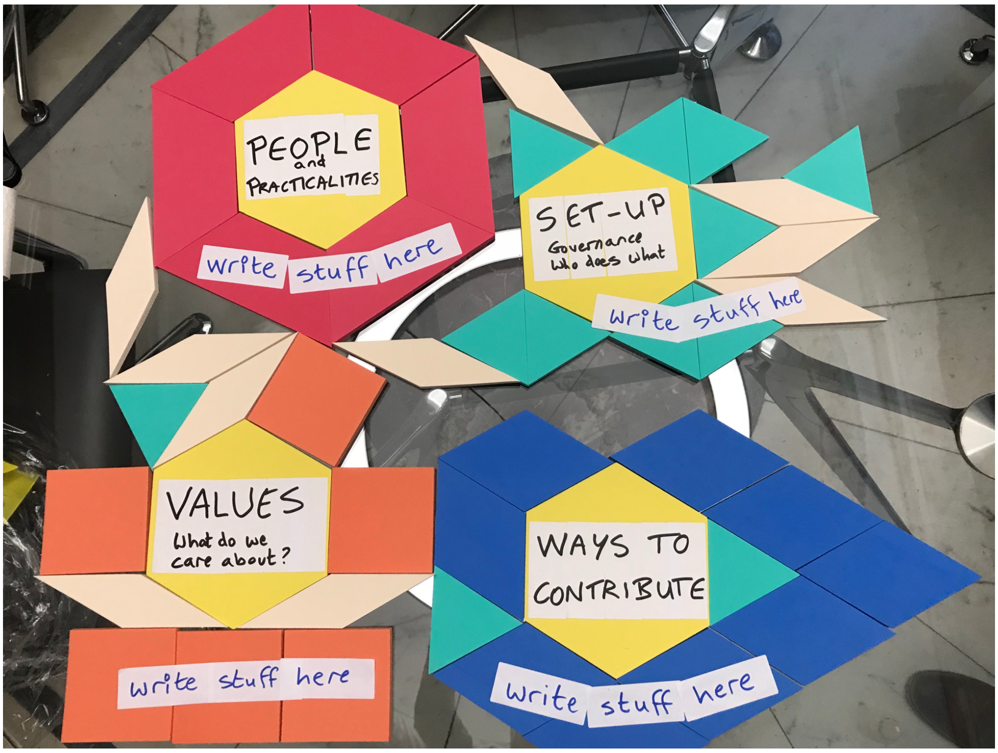

The group should **begin** by brainstorming on the question "what is good about what we do now?", and then cover "what could we do to strengthen this aspect of our PPI work?".

The group may wish to arrange the shapes into an object such as a tree, a building or even an animal. Or just make a random shape.

3. As the framework develops, people should walk around among the groups and exchange explanations for the themes they contributed to. For example:
   - suggest how to modify or improve other themes
   - discuss any parallels or contradictions between themes
   - link different themes together
   - clarify how the ideas for each theme would be implemented

TEA/COFFEE BREAK 30 minutes

GROUP TASK 2 25 minutes

Refining the framework

The next step is to prompt the groups to ensure that they have considered all the areas that are key to successful PPI. They don't have to add something if they don't think it's relevant. Below is a brief checklist, for the facilitator to bear in mind and use as a prompt, of ideas that have come out of the literature.

UNDERPINNING VALUES (for example)	
Commitment to building PPI together, through co-design and co-learning	☐
Recognise the patient experience as central	☐
Involve and support everyone equally, with attention to 'seldom heard' groups	☐
Mutual respect; researchers and lay partners respect each others' roles and perspectives	☐
Keep people informed; researchers and lay partners are clear and open about involvement in the research	☐
SET-UP AND PRACTICALITIES (for example)	
Governance; steering group, oversight, leadership	☐
Key roles and responsibilities including agreeing who the link person will be.	☐
Decide where meetings will be held (think about accessible venues for lay partners).	☐
Agree how and when participants will be paid (honorarium and expenses).	☐
Find out about training for lay partners and researchers and organise.	☐
Evaluation plan: decide how to measure the impact of PPI and who will do it.	☐

PEOPLE AND RELATIONSHIPS (for example)	
Keep in touch / keep people on board	☐
Ensure that everyone can contribute in they way they wish to	☐
Ensure that people are 'looked after' where needed (e.g. to take account of a physical or mental health condition)	☐
Manage conflict (which can sometimes be productive)	☐
HOW TO CONTRIBUTE TO RESEARCH (for example)	
Setting the research agenda (before a specific study is planned)	☐
Writing grant applications (including helping define what a 'good' outcome is)	☐
Writing recruitment information (invitation letters, participant information sheets and consent forms)	☐
Recruiting participants to a study	☐
Collecting and analysing data	☐
Disseminating findings e.g. writing lay summaries, giving talks, contributing to publications	☐

TASK 3

30 minutes

Plenary: finishing the framework and next steps

1. Each group '**show and tell**' their section of the framework. The whole group should discuss (and, hopefully, agree) each section and relate it to the wider framework.
2. When the framework is finished, take a **picture** of it (this might, for example, go on a poster or website).
3. Go through each main theme (central shape) and make a list of **what** needs to be done **by whom** to take this work forward.

For example:

- In UNDERPINNING VALUES, is there work to be done to engage seldom-heard groups or work towards a more democratic ethos between patient and lay partners, clinicians and researchers? What would this entail in practice, and who will do what?
- In SET-UP AND PRACTICALITIES, are the key structures and leadership in place and key roles allocated? Do training courses need to be accessed or developed, do (more) people need to be encouraged to attend these? If so, who will find out about courses, who will think about whom to invite, who will contact them?
- In PEOPLE AND RELATIONSHIPS, do patient and lay partners already feel well supported and in touch with the research group? If not, what do they feel would help? Who will be responsible for setting this up? On what timescale, and how will they let people know when it has been done? Are there areas of conflict (potentially productive) that need a facilitator to work through?

- In HOW TO CONTRIBUTE, are patients and lay partners currently included at all stages of the research? If not, what needs to be done to extend PPI to more aspects of the study (or future studies)? Who will lead on this?

NB the list of tasks also needs to include a task about following up: when does each item on this list need to be done, and who will check to make sure it's happened?

4. Think about **evaluation** of the group's PPI activity. How will you demonstrate impact? What would you like to measure? How?

For example:

- Numbers (on steering group, attending meetings, active on email list)
- Diversity (evidence that PPI has gone beyond the 'usual suspects')
- Training (what was provided, who attended)
- Surveys (patient / lay perceptions of the quality of their involvement, and researcher perceptions of the value provided by PPI contributors)
- Case narratives of good practice (where PPI went well)
- Significant events (where things didn't go so well, with learning points)
- Closing the loop (evidence that all the above fed into the research and its outcomes).

5. Finally, consider when the group would like to meet again and agree who will organise this. Subsequent meetings will not have a facilitator, so participants need to leave the workshop feeling that they can take their plan forward on their own.

Summary of the session

10 minutes

Facilitator to summarise
- the decisions made
- who is responsible for what action in operationalising the framework
- who is responsible for what action in evaluating the framework
- next meeting and who will organise

Note for NIHR BRC facilitators: Mention to participants (and repeat in a follow-up email) that the frameworking exercise and any follow-up activities will be reported to NIHR in the annual report. NIHR expects that as part of evaluation, participants should be making a note of PPI activities and their impact, and bringing these to the attention of research staff with a PPI role.

Thanks and close

If necessary, facilitator to offer to produce a summary of the meeting, perhaps with a synthesis of themes covered and/or a set of minutes? Process payment forms etc.

A note on the facilitator role

As facilitator you are a 'neutral player' – it is vital that you don't contribute to the content of the discussion or try to influence what is happening.

1. Ensure that everyone understands the tasks
2. Ensure that the environment is conducive to open dialogue (people don't talk over each other etc.)
3. Watch out for use of jargon; support people to ask for explanations and record them on a "Jargon Busting sheet" (flip chart paper on the wall).
4. Manage the more dominant group participants (asking them to wait to make their point, purposefully asking group members to contribute in turn etc.)
5. Support participants who are contributing less than others; ensure that you create space for them, keep eye contact with them to give them non-verbal cues to contribute.
6. Check to see if everyone can read what is being written on the foam shapes and prompt cards, read them out for clarity.
7. Keep the discussion and debate focused on the PPI framework, not other related issues.
8. Ensure that as participants come up with ideas, they also think about implementing them (Who will take the lead on this? What do they need to do? When?).
9. Ensure that as decisions are made, there is agreement within group to move on to the next activity.
10. Clarify and reflect important statements and decisions back to the group.
11. Note the areas of discussion where consensus has not been achieved, and why.

Brief for 'Values' section

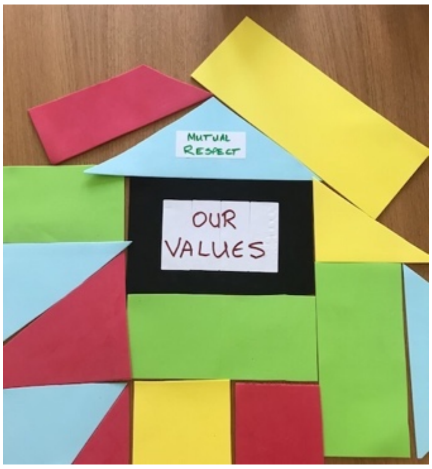

YOUR TASK:

Make a visual map to represent the important overarching VALUES (ideals and principles) that should guide patient and public involvement in research.

Use only one or two 'headline' words per shape – and write big!

SOME QUESTIONS TO SET YOU THINKING

For patients and lay people:

- Why do we want to be involved in research?
- How do we want to be treated?
- How do we want to work together with researchers?
- What has actually happened (good or bad) in the past?

For researchers:

- Why might it be a good idea for patients and lay people to be involved in research? What might the downsides be?
- What key principles could we follow to make patient/lay involvement more meaningful and productive for everyone?
- How do we think patients and lay people could best make a contribution?

For both:

- What would good look like in an ideal world? What should we avoid?
- Who should hold the power? In other words, how democratic should our patient and lay involvement be?
- Do any of the values or principles clash?
- How should we structure our work together to make sure we can actually act on these values?

OVER TO YOU…

Brief for 'People and relationships' section

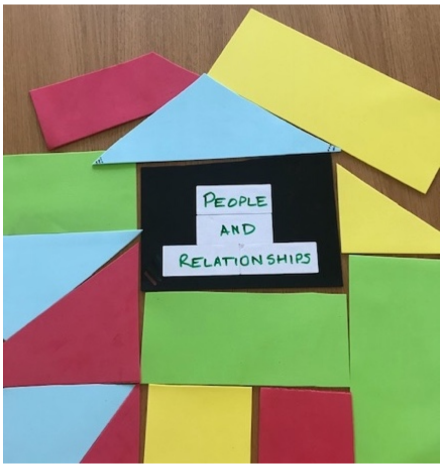

YOUR TASK:

Make a visual map to represent the important issues around people and relationships when patients and lay people seek to get involved in research.

Use only one or two words per shape – and write big!

SOME QUESTIONS TO SET YOU THINKING

For patients and lay people:

- What 'people issues' (positive or negative) come up when making our contribution to research?
- What might help build productive relationships – and what might get in the way?
- What kinds of support (broadly) would help us fulfil our role?
- What do we want researchers to tell us – and how?

For researchers:

- How do we currently go about relating to our patient and lay partners?
- What media do we use to get/keep in touch patients and the public – and could we be more creative?
- How do we currently support patients and the lay public? Which aspects of this support works – and which could be improved?

For both:

- Who is not in the room? How might we build better links with under-represented groups?
- How can we develop our relationships and strengthen them?
- What plans or structures do we need in order to help us put these ideas into practice?

OVER TO YOU…

Brief for 'Set-up and practicalities' section

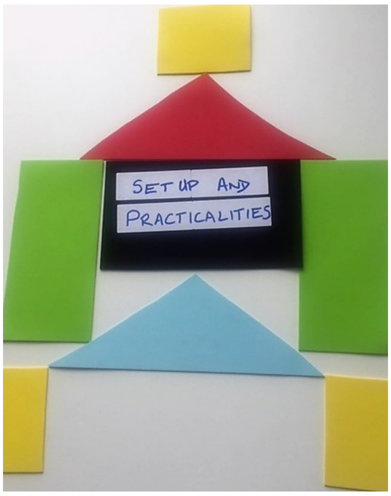

YOUR TASK:

Make a visual map to represent the various practicalities when patients and lay people get involved in research.

Use only one or two words per shape – and write big!

SOME QUESTIONS TO SET YOU THINKING

For patients and lay people:

- What are the practical issues that need to be sorted when you are contributing to a research study (or seeking to do so)?
- Which aspects of the practicalities tend to go well – and which aspects need a re-think?
- Has anyone got a story to tell from which we could learn?

For researchers:

- What governance arrangements and infrastructure do we have in place for addressing the practical aspects of patient and lay involvement?
- Whose job are these practicalities? If everyone's or no-one's, how might we ensure they get done? Who is actually *leading* on PPI?

For both:

- If we were writing the job description of a 'patient and public support person' for our research, what would we put on it?
- What would a really good system for supporting PPI in our research feel like (for both researchers and patients)?
- What plans should we make, and what roles should we allocate, to help us move forward with these ideas?

OVER TO YOU…

Brief for 'How to contribute' section

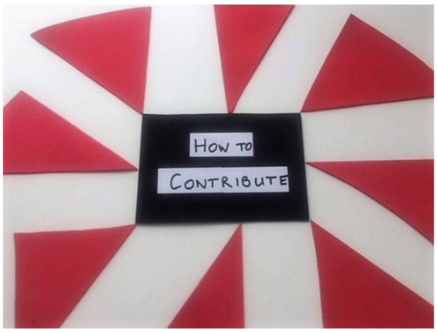

YOUR TASK:

Make a visual map to represent all the different stages and aspects of research where patient or lay input might be valuable.

Use only one or two words per shape – and write big!

SOME QUESTIONS TO SET YOU THINKING

For patients and lay people:

- What kinds of involvement have you had with research so far?
- Starting from the beginning (even before the research study has been thought of) and going through to after it ends, how might patients and lay people be involved at each stage?

For researchers:

- Can you draw a diagram of the different phases of your research study?
- Are patients already involved in every stage?
- Are they involved as much as you/they would like?

For both:

- Can you conceptualise an 'end to end' patient/public involvement plan?
- How detailed should such a plan be?
- Is PPI desirable or useful at every stage? If not, where should it be focused?
- What plans or structures do we need in order to facilitate involvement at all the stages where we think it is desirable?

OVER TO YOU…
